# Concentrated Load Capacity of Prestressed Hollow Core Floors: Research Based on Full-Scale Experimental Investigation

**DOI:** 10.3390/ma19204279

**Published:** 2026-10-10

**Authors:** Miłosz Jeziorski, Wit Derkowski

**Affiliations:** 1 Consolis Group Technology Development Centre, 90-527 Lodz, Poland; 2 KUT Doctoral School, Faculty of Civil Engineering, Krakow University of Technology, 31-155 Krakow, Poland; 3 Chair of Reinforced Concrete and Prestressed Concrete Structures, Krakow University of Technology, 31-155 Krakow, Poland; wit.derkowski@pk.edu.pl or wit.derkowski@lnu.se; 4 Department of Building Technology, Linnaeus University, 35252 Växjö, Sweden

**Keywords:** hollow core, precast concrete, precast floors, prestressed concrete, load distribution

## Abstract

**Highlights:**

- HC floors behave differently from single HC subjected to concentrated loads;
- Floors can withstand much higher loads than single elements;
- The increased stiffness of the entire floor system results in brittle failure mode;
- The observed floor behavior varied between the investigated HC cross-sections.

**Abstract:**

The geometry of precast prestressed hollow-core cross-sections, production-induced variability in thin-walled concrete properties, and the use of exclusively longitudinal strands as reinforcement present challenges in designing hollow-core floors under concentrated loads. These loads induce complex stress states in webs and flanges, largely governed by concrete tensile strength. While individual hollow-core units have been widely studied, experimental data on full floor systems remain limited despite their expected higher load capacity. To address this gap, an experimental program was conducted on single elements and full floor systems subjected to concentrated loads up to failure. Clear differences in behavior were observed. Single elements exhibited ductile failure governed by shear and flexure, whereas full floor systems failed in a brittle manner dominated by torsional and transverse effects. Interaction between adjacent units and tying systems increased the load capacity relative to isolated elements; however, the magnitude of this enhancement depended on the specimen and load location, with enhancement ratios ranging from 1.09 to 1.49 at edge positions and from 2.03 to 2.51 at central positions. Despite the brittle failure modes, the structural integrity was preserved. Differences were also found between material properties obtained from standard-sized and drilled core specimens. Nevertheless, numerical models based on standard specimen properties showed good agreement with experiments and were not significantly affected by variations in tensile strength, indicating that full floor systems are less sensitive to concrete tensile strength than single elements.

## 1. Introduction

The specific trapezoidal voided shape of the precast prestressed hollow core slab (HC) and its production technology (extrusion or slipforming), which eliminates the use of vertical and transverse reinforcement, makes HC one of the few commonly used building components in the construction sector that does not contain reinforcement other than longitudinal strands. According to the newest European design standard for concrete structures, EN 1992-1-1:2023, members such as lintels or other members of minor importance, which do not contribute to the overall resistance and stability of the entire structure, as well as solid, ribbed, or hollow core slabs where the transverse redistribution of loads is possible, can be designed without shear and torsional reinforcement. In contrast to other elements mentioned in the design standards, HC is often used to construct floors of considerable spans (even up to 20 m), complex geometries, and loading conditions. HC floors are also considered as diaphragms that contribute to the overall stability of the structure. The reduced material consumption associated with the hollow core geometry and production technology results in a lower environmental impact compared to conventional flooring systems [1,2,3,4]. As prestressed precast elements, HC units are also inherently suitable for reuse, supporting circular construction principles [5,6]. These characteristics contribute to their widespread application and sustainable performance.

However, the lack of vertical and transverse reinforcement as well as the specific shape of the HC cross-section introduces considerable challenges and risks, as the shear, torsional, and transverse flexural capacity are largely governed by the uniaxial tensile strength of concrete [7,8,9,10,11,12,13,14,15,16,17,18]. The shear–tension capacity *V_Rd_*_,*c*_, is obtained from the transformed principal stress equation, as given in the European design standard for hollow core slabs [19] in Equation (1). Similarly, torsional capacity *T_Rd_*_,*c*_ is expressed in Equation (2). The formulation is further extended to account for the combined shear–torsion interaction, expressed through *V_Rd_*_,*c*,*T*_, as given in Equations (2) and (3), respectively [7]. The concentrated load applied to HC shall, in accordance with [19], be limited to a value *F_d_* given by Equation (4), which is the product of the tensile strength and the section modulus of the upper or lower flange. Furthermore, the capacity is further limited due to the punching shear *V_Rd_*_,*p*_ and joint *V_Rd_*_,*j*_ capacity as given in Equations (5)–(7).

(1) $$V_{\mathrm{Rd},c}=\frac{{\mathrm{Ib}}_{w}\left(y\right)}{S_{c}\left(y\right)}\left(\sqrt{f_{\mathrm{ctd}}^{2}+\sigma_{\mathrm{cp}}\left(y\right)f_{\mathrm{ctd}}}-\tau_{\mathrm{cp}}\left(y\right)\right)$$

(2) $$T_{\mathrm{Rd},c}=W_{T}f_{\mathrm{ctd}}\sqrt{1+\frac{\sigma_{\mathrm{cp}}}{f_{\mathrm{ctd}}}}$$

(3) $$V_{\mathrm{Rd},c,T}=\left(1-\frac{2b_{w,\mathrm{out}}}{\sum b_{w}}\frac{T_{\mathrm{Ed}}}{T_{\mathrm{Rd},c,\mathrm{web}}}\right)V_{\mathrm{Rd}.c}$$

F_d_ = 3 W_L_ f_ctd_ (4)

(5) VRd,p=beff h fctd(1+0.3ασcpfctd)

V_Rdj_ = v_Rdj_(a + h_j_ + h_t_ + 2a_s_) (6)

(7) $$v_{\mathrm{Rdj}}=\min\left(0.25f_{\mathrm{ctd}}\sum h_{f};0.15f_{\mathrm{ctd},j}h_{j}\right)$$

where:

*I*—HC sections’ second moment of area;

*b_w_*(*y*)—width of HC cross-section at the chosen point along the vertical (y) axis;

*S_c_*(*y*)—first moment of the area above y height and about the centroidal axis;

*σ_cp_*—normal stress component due to prestressing and applied loads;

*f_ctd_*—design tensile strength of concrete;

*τ_cp_*—shear stress due to the prestressing force gradient in the prestressing transfer zone;

*W_T_*—torsional modulus of HC section with respect to the web, or upper or lower flange;

*b_w_*_,*out*_—width of the outermost web measured at the center of gravity;

*T_Rd_*_,*c*,*web*_—torsional moment capacity calculated about the outer web;

*W_L_*—transverse modulus of HC section with respect to either top or bottom fiber;

*b_eff_*−effective width of webs, which depends on the loaded area and the load location;

*h*—height of the HC cross-section;

$\alpha =\frac{l_{x}}{l_{\mathrm{bpd}}}\leq 1$—strands anchorage factor;

*a*—length of the load parallel to the joint;

h_j_—height of the joint;

h_t_—height of the topping;

*a_s_*—distance between the center of the load and the center of the joint;

Σ*h_f_*—sum of the smallest thickness of the upper and lower flange;

*f_ctdj_*—design value of the tensile strength of the joint concrete.

The concrete mix used for HC is distinct from conventional mixes. The requirements imposed by the production process such as the density, tight compaction, stability immediately after forming, high early strength, good bond to the prestressing tendons, and low rheological deformability [20,21] lead to the use of a very dry, high-strength concrete with a water-to-cement ratio of 0.30 to 0.40 [22]. The size of the elements, thin walls, the properties of the concrete mix, and the curing process result in substantial differences between the tensile strength and elasticity of concrete in HC and the corresponding properties of concrete assigned to a specific class [21]. This issue, together with the brittle failure mechanism governed by the tensile strength of concrete, raises concerns regarding the reliability of HC floors’ performance.

The issue of HC shear failure has gained much attention in research over the last decades [8,9,10,11,12,17,18,23,24,25,26,27], notably because it is relevant to the majority of both regular and complex HC applications. The problem of the torsional and transverse flexural capacity of HC does not concern regular floor layouts subjected to uniformly distributed loads; yet, such cases are rare in floors of modern buildings, which are often characterized by irregular shapes, or interruptions caused by openings required for building systems, or are subjected to differential, often concentrated, loads. In such cases, the capacity of HC floors can be governed by a complex stress state in HC webs and flanges, induced by a combination of flexure, shear, torsion, and transverse effects. In contrast to shear, research addressing the aforementioned problems remains limited. The problem of torsional and the combined shear–torsional behavior of single HC elements subjected to concentrated loads was studied both experimentally and numerically by Broo and Pajari [7,14,28,29,30], while numerical studies on torsion have been performed by Sarkis [13]. The transverse flexural behavior of hollow core strips was studied by Pisanty [15], while experiments on single slabs subjected to point loading were performed by Aswad and Jacques [31], and McGinnis [25], as well as Lucio and Castilho [32].

The experiments on single slabs do not fully reflect the actual behavior of full floors. Due to the presence of the cast on the site joints and the torsional stiffness of individual HC units, the concentrated load applied to an individual unit can be further distributed [19,33], and the restraints imposed by supports or adjacent structural elements [34,35,36] can further improve the load-bearing capacity of full floors. It is therefore anticipated that the concentrated load applied to the floor can be significantly increased, compared to a single element, thereby supporting a more resource-efficient and sustainable structural design. The aforementioned behavior, often referred to as load distribution, can be taken into account in the design using simple graphical methods [19,37] or analytical models [38,39], which can nevertheless lead to contradictory results when directly compared [33,40], despite sharing similar assumptions. The methods and models are also based on the theory of elasticity, which is not usually valid for the ultimate limit state (ULS) design. In contrast to single-element tests, experiments on full floors are sparse, often poorly documented, outdated, and characterized by a significant scatter of adopted parameters [33]. Out of the experiments mentioned in [33], only four studies [41,42,43,44] considered concentrated loads leading to failure. The well-documented experiments [41,42,44] on similar 400 mm-deep HC cross-sections were studied numerically by Broo, and the authors’ numerical analysis of these experiments, accompanied by parametric studies on the 400 mm cross-section [45], showed that the HC floor concentrated load capacity can be significantly improved and is often substantially different from the single element behavior. Furthermore, the comparative analysis of existing design standards and the results of the aforementioned tests [46] showed that the code prediction can often lead to underestimated capacity results. Further numerical studies performed by the authors on three different HC cross sections [47] showed considerable differences in HC behavior governed by the shape of the cross-section. However, given the sparse experimental evidence on the concentrated load-bearing capacity of full HC floors, robust conclusions and design recommendations could not be drafted.

This motivated the authors to design and execute a full-scale experimental program on three hollow core floors, accompanied by single-element tests and material tests. The floors were tested in two phases. In the first phase, concentrated loads were applied in various positions and kept relatively low, and the main objective of this phase was to study load distribution in linear elastic conditions. A detailed description of this phase as well as its results can be found in [48]. The second phase, discussed herein, considered failure conditions, in which floors were loaded until failure in two chosen positions. In contrast to previous experiments where single point loads were applied at the midspan of the floor [41,42], the loads were applied to two or three webs, similarly to [43,44] where loads were applied in support regions, to avoid possible local effects. The full floor tests were accompanied by tests performed on single-element specimens to compare the failure mechanism and bearing capacity with those observed in full floors. This way, the contribution of the adjacent elements to the overall capacity of the whole floor assembly was assessed and quantified, giving valuable insight into the behavior of full precast floor systems under concentrated loads in post-elastic conditions.

In contrast to the previous test conducted by Pajari [44] on a 400 mm deep hollow-core floor, in which loads were applied in the support regions to investigate the interaction between shear and torsion, the test setups considered in the present study employed eccentric loading at the floor midspan, where additional flexural effects become significant. As reported by Broo [28], no substantial redistribution of shear and torsion was observed in the aforementioned experiments, despite the floors exhibiting a higher load-bearing capacity than individual specimens. Conversely, the authors’ previous work concerning the first phase of the experimental program [48] demonstrated that, for midspan load positions, load redistribution is significant. Unlike two earlier studies on HC floors composed of similar 400 mm-deep elements [41,42], in which loads were applied over a single web and resulted in local failure modes, the experiments presented herein employed load arrangements similar to those used by Pajari [44]. This approach enabled the investigation of a possible shift in the governing failure mechanism and a further increase in the load-bearing capacity, as predicted by the authors’ numerical simulations [45]. Furthermore, a precise assessment of the load-bearing enhancement achieved in the floor systems investigated in [41,42] was not possible due to a fact that no analogous single-element test were performed. Accordingly, the present research provides a novel perspective on the point-load-bearing capacity of HC floors with different cross-sections subjected to combined shear, bending, and torsion.

The primary objective of this work is to investigate the behavior of point-loaded hollow core floors through full-scale experimental testing. The experimental results obtained from full-floor specimens are subsequently compared with those from corresponding single-element tests to quantify the potential enhancement in load-bearing capacity and to identify changes in the governing failure mechanism. The study also aims to determine the mechanical properties of concrete using both standard-sized and drilled core specimens which represent the possible size and production method effect on the mechanical properties of concrete within the actual element. The experimental results, including the recorded load–displacement responses, measured mechanical properties of concrete, and observed crack patterns, are validated through a nonlinear finite element analysis to provide further support for the interpretation of the experimental observations.

The obtained results are also briefly compared with the provisions of the relevant European Standard, with particular attention given to the discrepancies between the experimental findings and the standard predictions and to the accuracy of the latter in estimating the load-bearing capacity. However, the work presented herein is not intended to provide simplified engineering approximations of the observed phenomena. Rather, it constitutes a valuable, novel contribution towards their development by providing an extensive and experimentally validated dataset that may serve as a basis for further research and the development of practical design approaches.

The scope of the experimental investigation imposes several limitations on the generalization of the obtained results. The experimental program comprised three full-scale floor specimens and three corresponding single-element tests, representing three different hollow-core cross-sections. The assessment of the point-load-bearing capacity under failure conditions was further limited to two load positions. In addition, the investigation considered monotonic, short-term loading under ambient temperature conditions and did not address other loading aspects such as cyclic or dynamic loads, fire conditions, or long-term effects. Consequently, the findings should be interpreted within the range of cross-sections, loading configurations, and test conditions investigated.

## 2. Methods

### 2.1. Experiments on Single Elements and Full-Scale Floors

**Figure 1:**
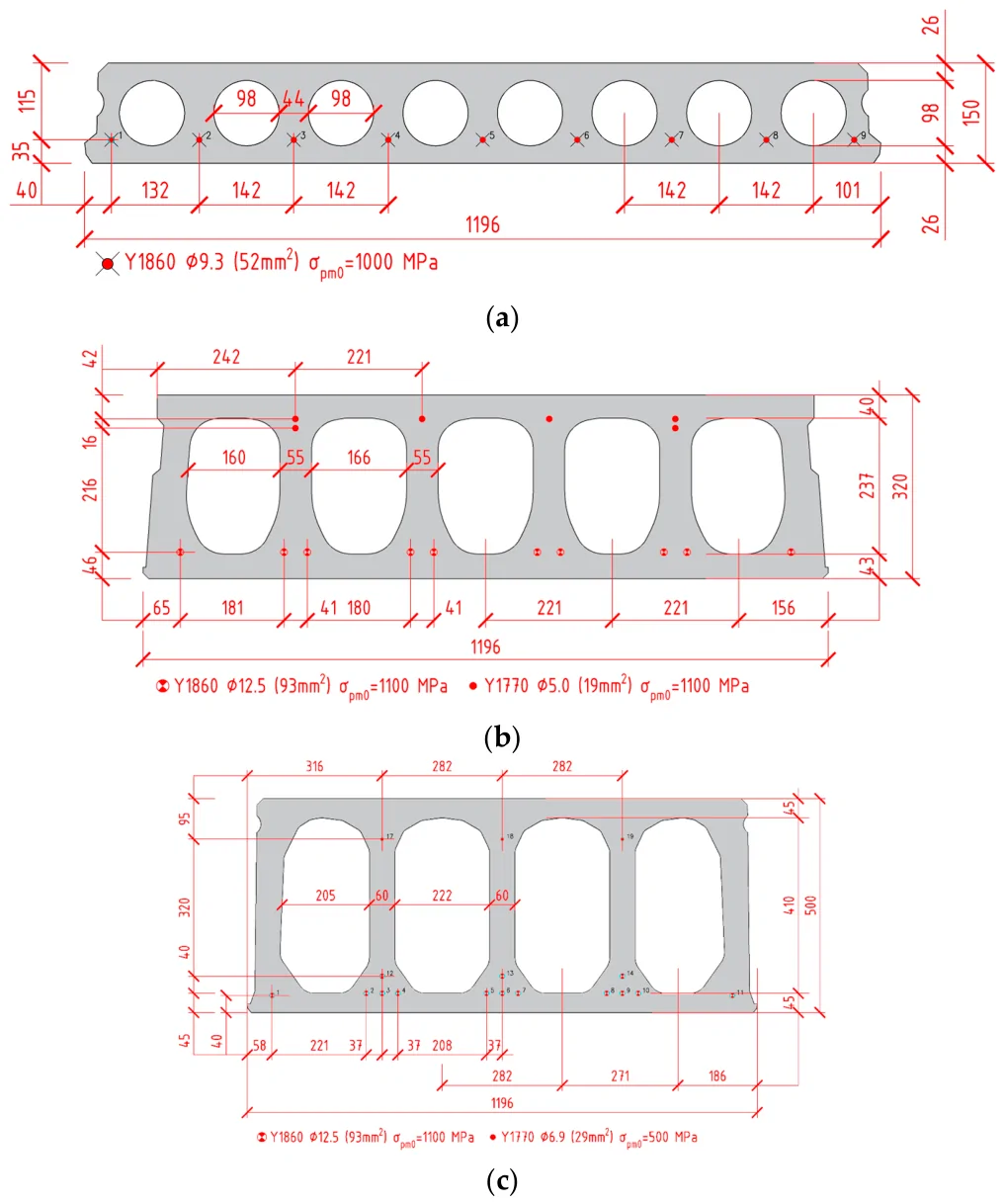
Cross-sections of HC elements used in the experiments: (**a**) SP150, (**b**) AVU320, and (**c**) HC500.

The full floor and single slab experiments were performed using three hollow core cross-sections produced by European manufacturers; the shape of the sections, prestressing reinforcement layout, and initial stressing are presented in Figure 1a–c. The chosen cross-sections represent commonly used shallow, circular-voided extruded section HC150 (Figure 1a), an average-depth, slip-formed rectangular-voided section AVU320 (Figure 1b), and the deepest section available on the market, allowed by European Standard [19]—extruded HC500 (Figure 1c). As can be seen in Figure 1, these are thin-walled sections, with wall thicknesses ranging from as little as 26 mm up to 60 mm, which can lead to additional scatter in the mechanical properties of the concrete.

**Figure 2:**
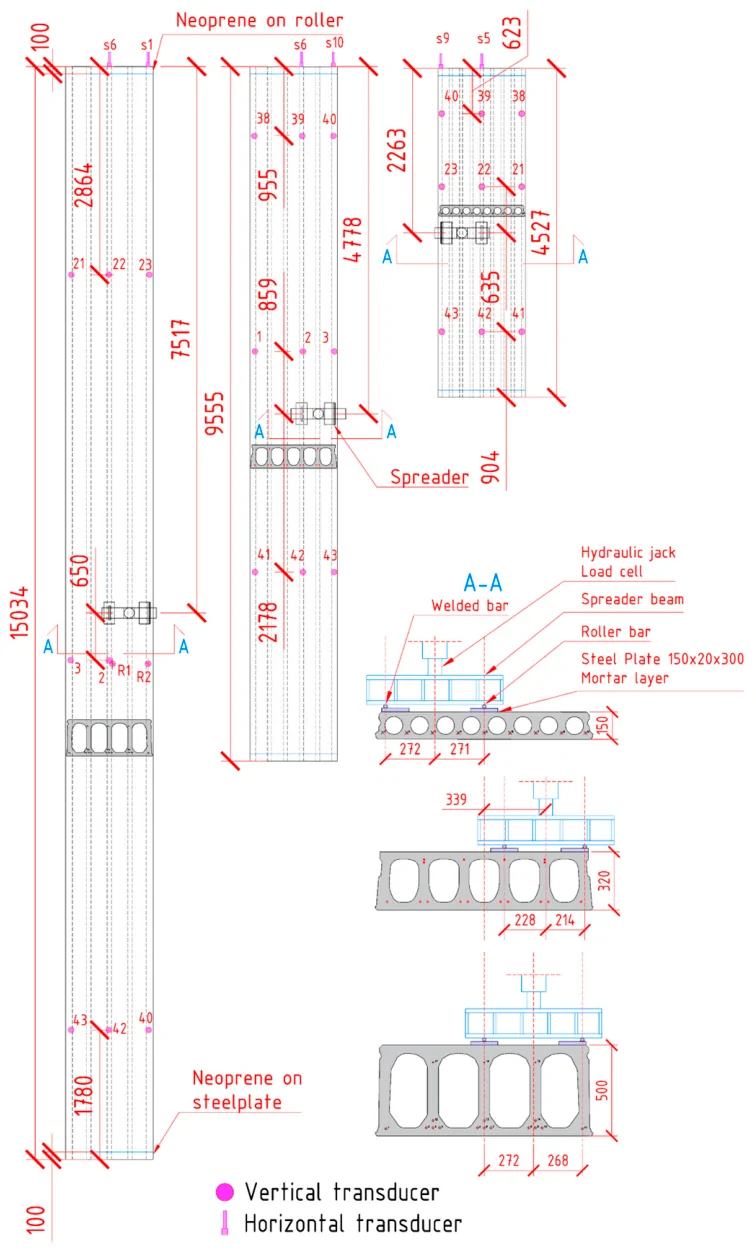
Sketch of the single-element tests’ setup.

The experimental program began with single-element tests. The elements measured 4527, 9555, and 15,034 mm in length in the case of HC150, AVU320, and HC500 sections, respectively. The spans were selected to maintain an approximately constant span-to-depth ratio of 30, representative of typical practical dimensions. Figure 2 presents the overall layout of the three single-element tests.

The specimens were supported through a 100 × 8 mm neoprene strip placed on a steel plate, which rested on a roller support on one side of the specimen. On the other side, the specimen was supported on a steel beam fastened to the laboratory floor. The load was applied similarly to experiments by Pajari [29,44]. Two steel plates measuring 300 × 20 × 150 mm were placed on the central and outer web of the specimens through a high-strength mortar layer. In contrast to previous experiments [29,44], where loads were applied in support regions, eccentric loading was applied in the midspan of the elements. A steel spreader beam was placed on the steel plates through two roller bars. The load was applied to the spreader through a force-controlled hydraulic jack attached to the loading frame, a load cell placed between the spreader and hydraulic jack provided constant force measurement. A set of LVDTs was used to measure the vertical displacements, and, additionally, horizontal transducers were placed against chosen strands to measure the relative slip. The load was applied to the specimen until failure.

**Figure 3:**
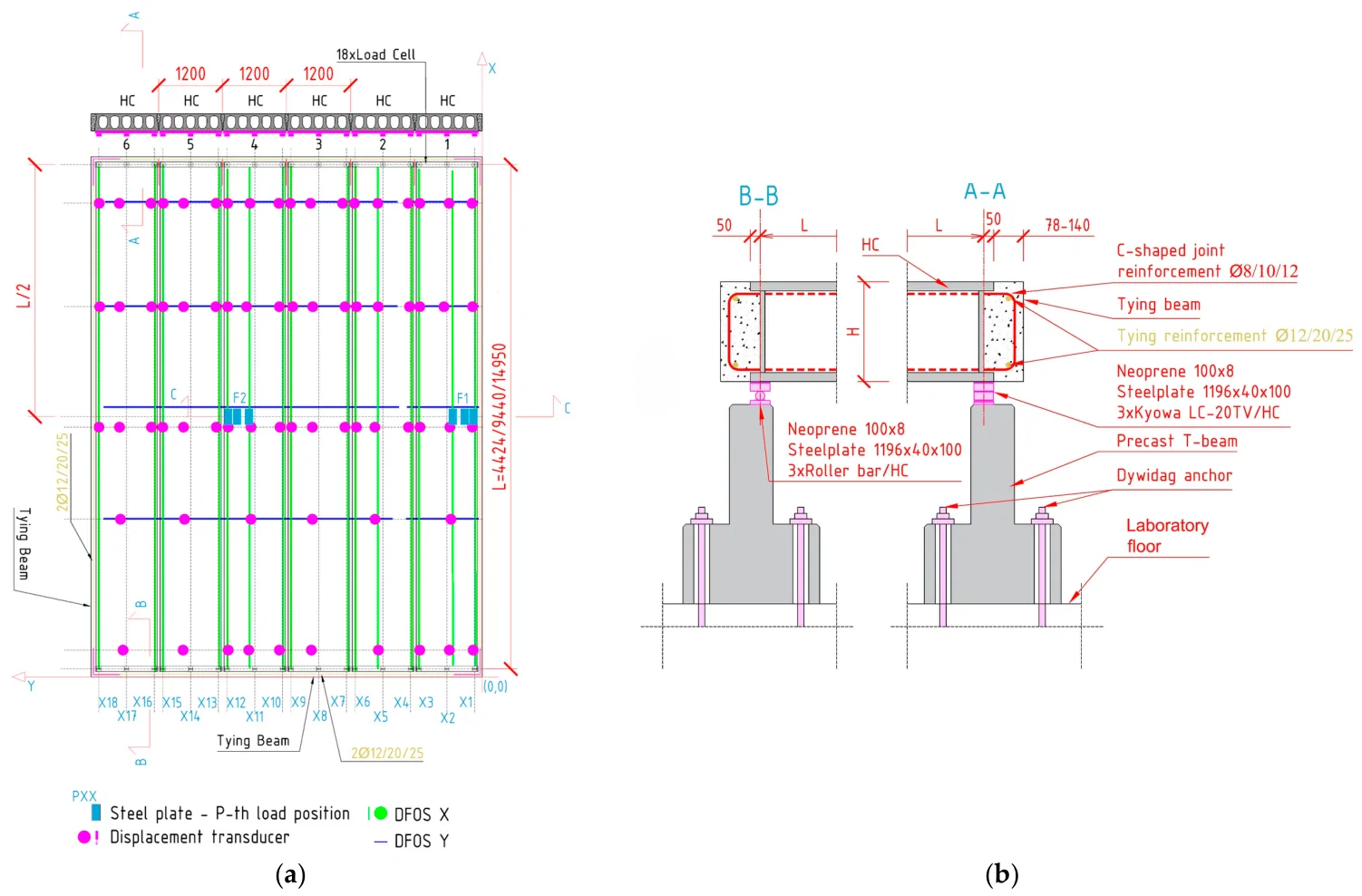
Sketch of the floor experimental setup: (**a**) plan view illustrating floor geometry and positions of measuring devices, and (**b**) sections illustrating support conditions.

Three full-scale floor tests were conducted. Each test was built of six identical HC units, HC150, AVU320, and HC500 in Floors 1, 2, and 3, respectively. The HC units were of the same span length L and from the same production cycle as the single elements, thereby ensuring the use of the same concrete mix throughout the entire experimental program. Figure 3 presents the typical geometry of the floor experiment together with measuring devices’ locations and cross-sections illustrating support conditions.

**Figure 4:**
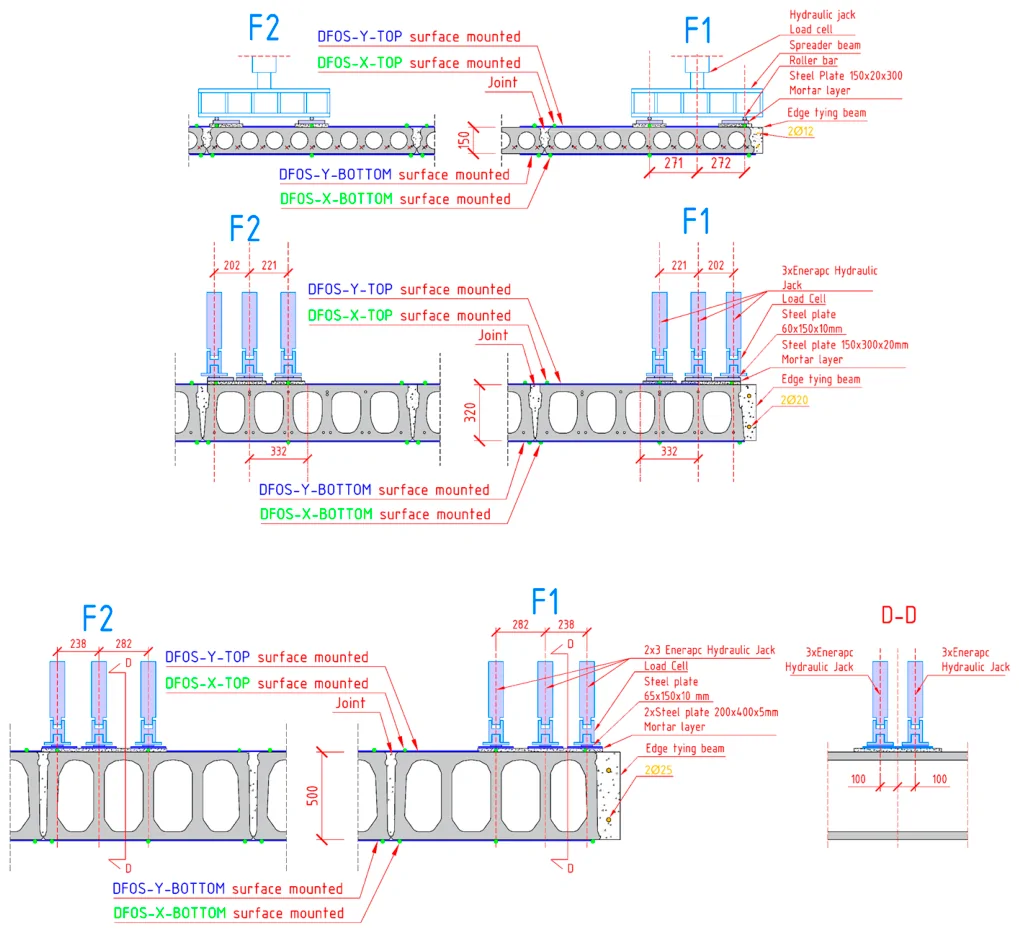
Sketch illustrating loading conditions in each experiment (cross-section C-C of Figure 3).

Each HC unit was supported by three identical load cells with spherical bearings or roller bars placed on inverted precast T beams—see sections A–A and B–B in Figure 3. Peripheral cast-on-site reinforced concrete tying beams were cast after the joints were grouted and mechanically pre-cracked. Displacement transducers attached to aluminum frames were used to measure vertical displacements. Distributed Fiber Optic Sensors (DFOS) glued to the top and the bottom surface of the floor in both longitudinal (X) and transverse (Y) directions measured strains. Further details regarding the preparation of the floor specimens, their geometry, and instrumentation can be found in [48]. Point loads were applied monotonically at two positions, first on the outer element (F1) and subsequently at F2. Load in Floor 1 was applied in a similar manner to the single-specimen experiments. In Floor 2, a set of three hydraulic jacks supplied by the same pressure line was used. In Floor 3, a set of six jacks was used—see Figure 4.

### 2.2. Evaluation of the Material Properties of Concrete

Material properties of concrete used to produce HC slabs were tested on samples delivered with the specimens by HC producers. Cube samples measuring 150 × 150 × 150 mm were used to test the concrete compressive cube strength *f_c_*_,*cube*_, and 300 × ⌀150 mm cylinders were used to test the compressive strength *f_c_*_,*cyl*_, the modulus of elasticity *E_c_*, splitting tensile strength *f_ct_*_,*sp*_, as well as uniaxial tensile strength *f_ct_* (for HC500 only). Prisms measuring 400 × 100 × 100 mm were used to test the flexural tensile strength *f_ct_*_,*fl*_ in a three-point bending test. Since the single-element tests were performed earlier than the full floor experiments, three cube samples were tested for each HC type in a compressive strength test in order to assess the difference in material properties of single slabs and full floors stemming from the age difference. Cube and cylinder samples were taken during casting of the joints and tying beams to evaluate the material properties in a similar manner to the HC concrete. Apart from tests on standard specimens, material properties of HC concrete were tested on cores drilled from the HC after floor disassembly. The samples were drilled from the top or bottom part of the undamaged webs, and samples were taken from the outermost and central elements (directly loaded slabs). Due to the specific shape of the HC cross-section, the size of the cores had to be limited to ⌀35 × 70, ⌀45 × 90, and ⌀45 × 90 in Floors 1, 2, and 3, respectively. The cores were tested for compressive strength *f_c_*_,*core*_, elasticity modulus *E_c_*_,*core*_, and uniaxial tensile strength *f_t_*_,*core*_. It should be noted that cores were taken after the floors had been disassembled, resulting in a significant difference in the age of the drilled samples and the standard samples.

### 2.3. Numerical Simulations

Since the experiments were, due to their scale, performed on a limited number of specimens, and, due to the fact that properties of HC concrete are strongly affected by the production process and size effects stemming from the thin-walled geometry, a nonlinear finite element analysis (NLFEA) was performed to validate the obtained experimental results and reinforce the conclusions of this study.

**Figure 5:**
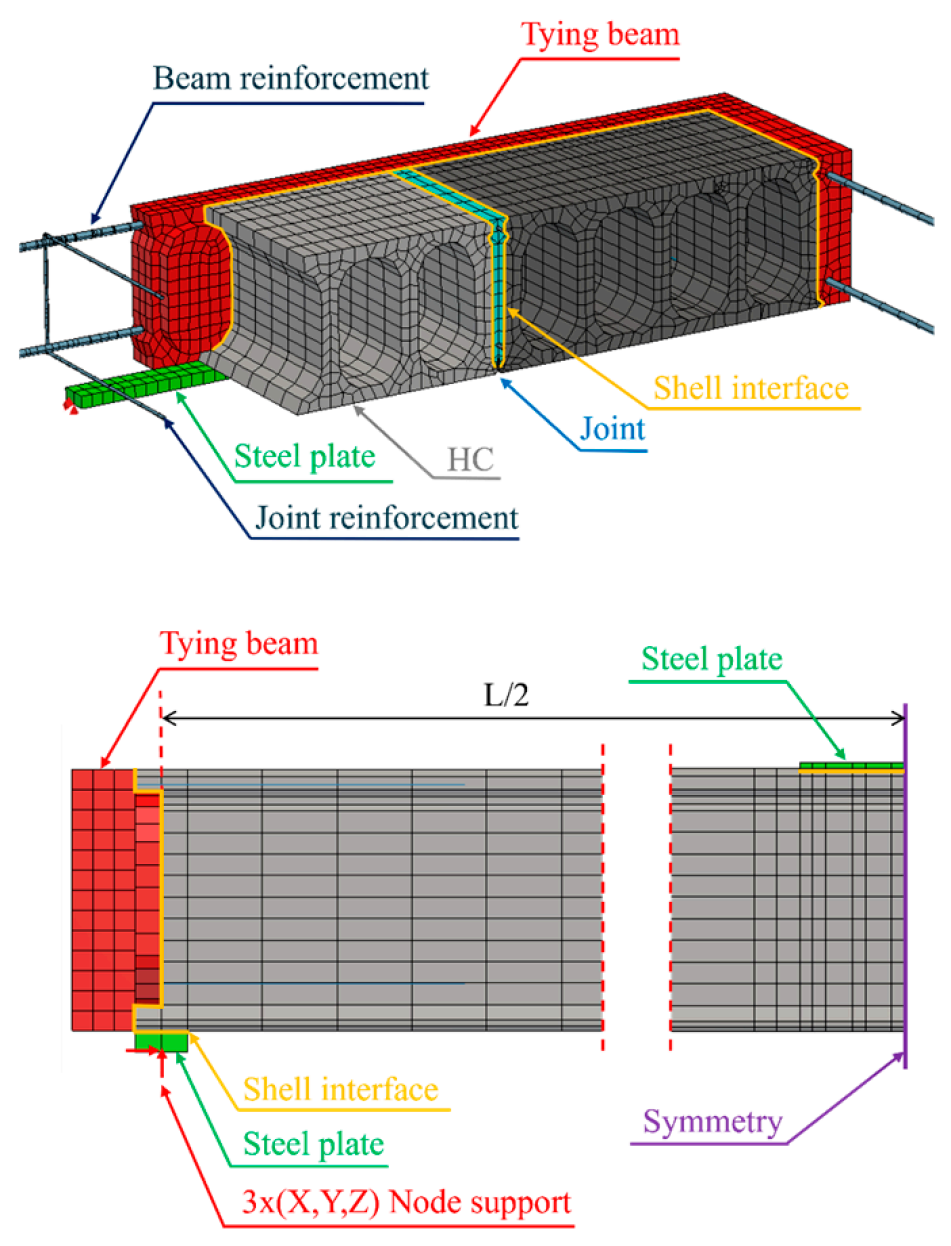
Finite element discretization of the numerical model—illustrated on the example of HC500 floor.

The numerical analysis is limited to full floor specimen models prepared in DIANA software. HC, joints, tying beams, and steel supporting elements were modeled with solid three-dimensional 20-node elements. Figure 5 illustrates the overall principle of the finite element discretization.

Coulomb–Mohr shell interfaces were applied between cast-on-site and precast elements, while a no-tension with shear stiffness reduction interface was applied between steel and concrete parts. Bar elements were used to model reinforcement in the tying beams as well as prestressing tendons. The roller bar and load cell supports were modelled as X, Y, Z nodal point supports. Since loads were applied in the midspan of the floor, a symmetry axis was used to limit the size of the model.

Bond slip of the tendons was included using the Khalaf and Huang model [49] with peak bond stress obtained from Orr et al. [50]. The Cairns [51] bond slip model was applied for prestressing wires, and bond of cast-on-site reinforcement was modelled according to Dörr [52]. A total strain rotating crack model was used, with Feenstra [53] parabolic model describing the compressive behavior of concrete. Peak compressive stress was assumed to be equal to 0.8 the mean *f_c_*_,*cube*_ value, since values measured on cubes were deemed the most accurate. The Vecchio and Collins [54] lateral cracking reduction model was applied together with the Selby and Vecchio [55] confinement model, while compressive fracture energy was defined according to Nakamura and Higai [56].

The tensile behavior of concrete was modelled with the Hordijk [57] softening curve. Since large deviations in the measured tensile strength of concrete were observed in Floors 1 and 3, two alternatives were considered. In the first, the tensile strength obtained from prism samples, recalculated to uniaxial tensile strength according to Model Code 2010 [58], was used; in the second, the mean tensile strength measured on drilled core specimens was applied. Tensile fracture energy was calculated according to Model Code 2010 [58]; Govindjee crack bandwidth specification [59] was applied.

The load was applied as a prescribed deformation of the central node of the loading plate; a tying set was applied between the loaded node and the surface of the steel plates that corresponded with the size of the loading apparatus. The numerical models did not include the actual loading sequence—instead, a separate model with undamaged elements was considered for the analysis of position F2.

The modelling approach followed the same principles as previously described in detail in [45,47,60], where the numerical models of 400 mm-deep floors [41,42,44] were considered, resulting in a very good fit with the existing experimental data. Therefore, further details of the numerical model can be found in above-mentioned publications.

## 3. Results

### 3.1. Experiments on Single Specimens

**Figure 6:**
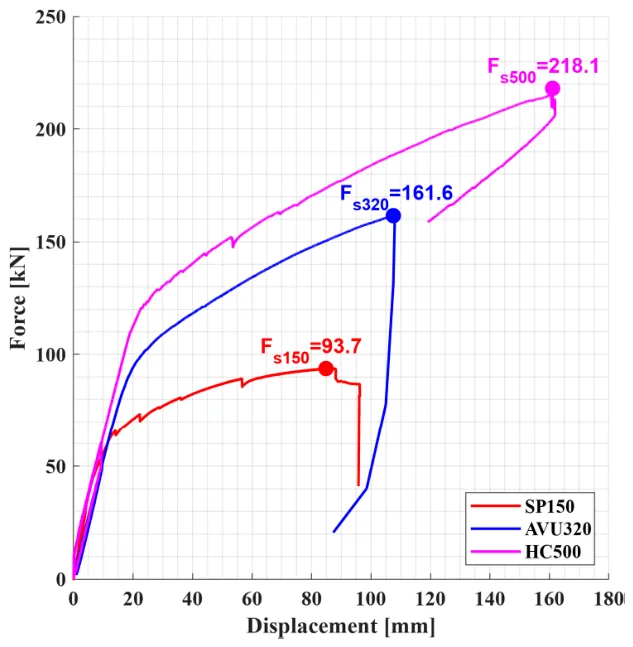
Load–displacement curves recorded in the single-specimen experiments.

The behavior of single specimens is illustrated on load–displacement curves obtained from selected transducers located closest to the loading plates—see Figure 6.

**Figure 7:**
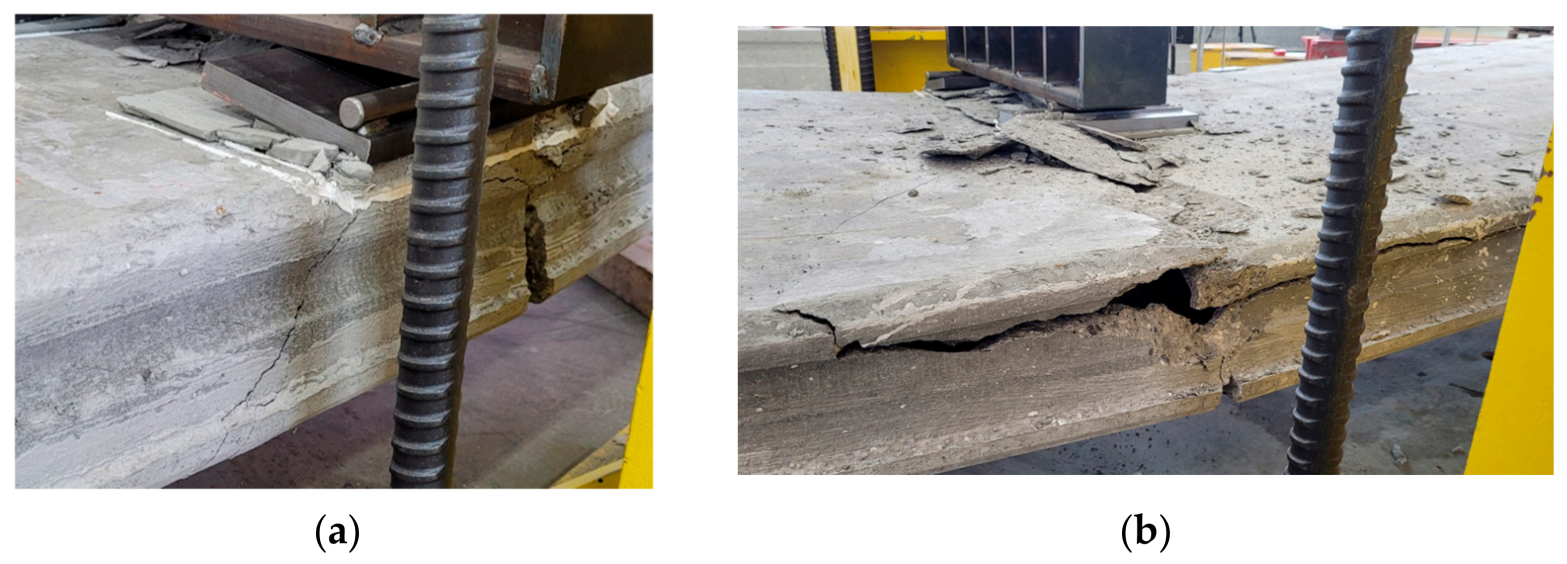
Cracks observed in single SP150 element: (**a**) after failure on the loaded ultimate web, and (**b**) after failure on the unloaded ultimate web.

In the SP150 experiment, a flexural crack was first observed on both sides of the specimen, shortly followed by an inclined crack on the side of the specimen closer to the spreader beam—see Figure 7a. Similarly, a flexural crack opened on the opposite side of the specimen—see Figure 7b. Upon reaching the load value of 93.7 kN, a sudden flexural-compressive failure occurred in the top part of the specimen.

**Figure 8:**
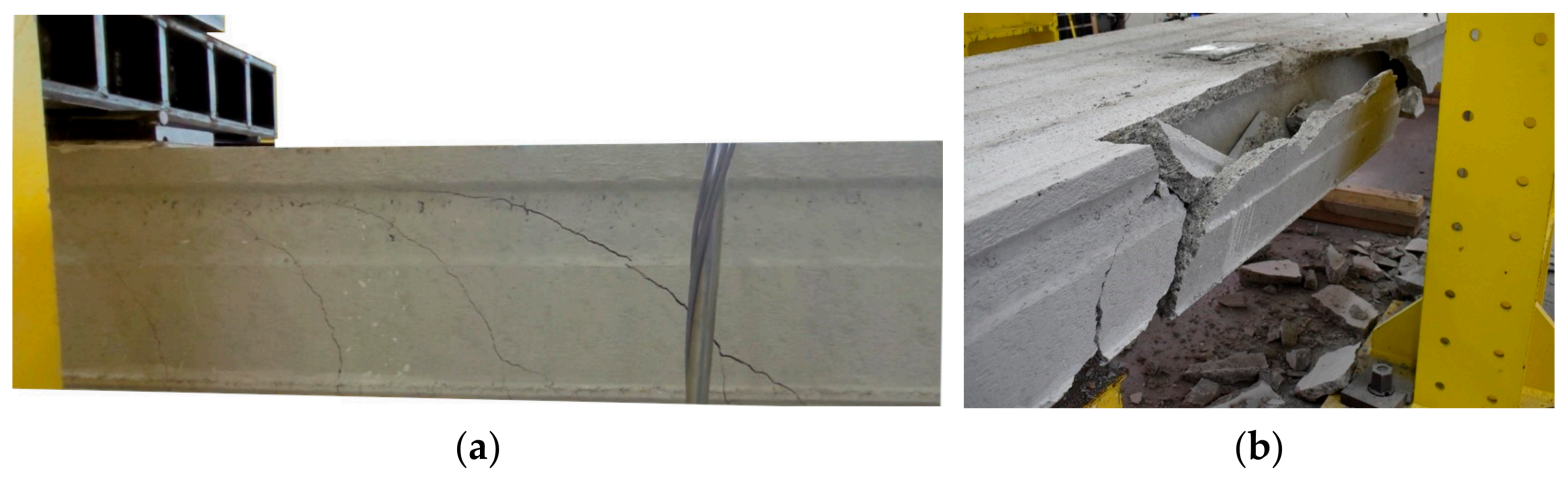
Behavior of the AVU320 element: (**a**) shear–flexural cracks observed on the outer web shortly before failure, and (**b**) damaged specimen photographed after the test.

Flexural cracks on the soffit of the AVU320 were observed first. The cracks propagated onto the outer, loaded web, and the vertical flexural crack turned into an inclined shear crack—see Figure 8a. The element failed at a load value of 161.6 kN in a very brittle, explosive manner. Damage was localized in the outer loaded web, and longitudinal cracking occurred in the top and the bottom flange causing a complete separation of the outer web from the rest of the specimen—see Figure 8b.

**Figure 9:**
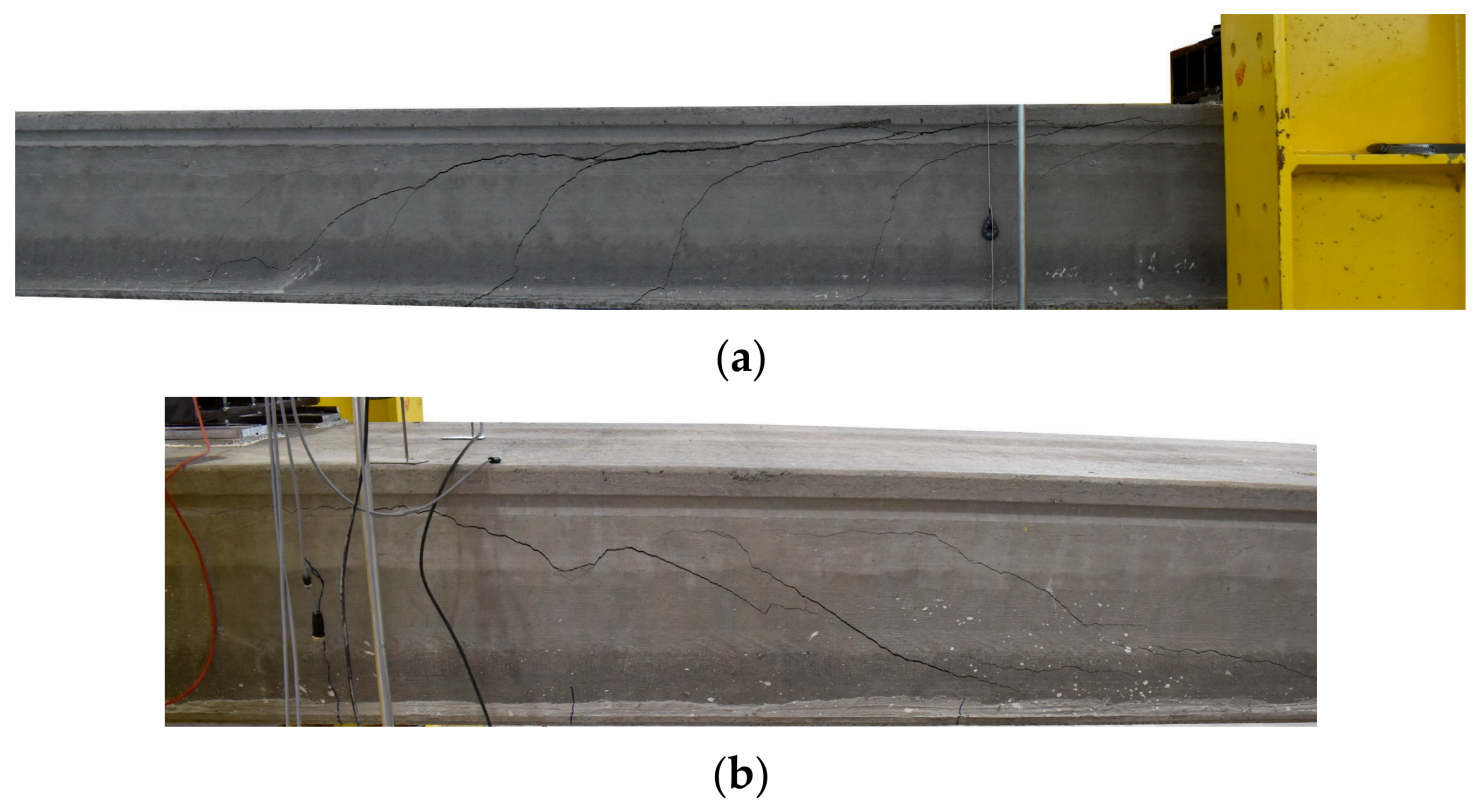
Behavior of the HC500 element: (**a**) shear–flexural cracks on the outer loaded web photographed after failure, and (**b**) inclined cracks on the outer unloaded web photographed after failure.

The behavior of HC500 was similar to AVU320. Flexural cracks were observed first on the bottom surface; the cracks propagated onto the outer, loaded web, and turned into inclined shear cracks—see Figure 9a. The element failed at 218.1 kN due to shear–flexure. Inclined cracks were also observed on the unloaded web—see Figure 9b.

### 3.2. Experiments on Full Floors

**Figure 10:**
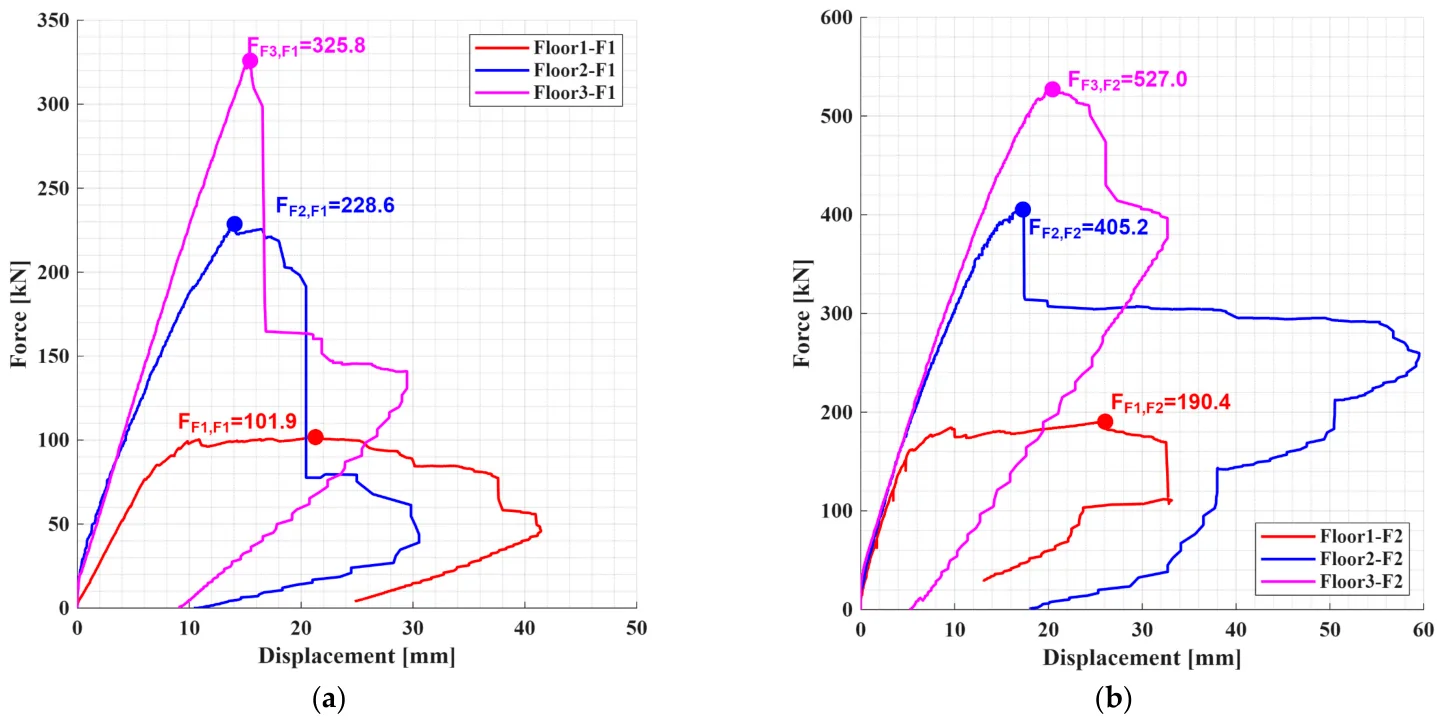
Load-displacement curves recorded in the full floor experiments: (**a**) load in position F1, and (**b**) load in position F2.

The behavior of full floor specimens is illustrated on load–displacement curves recorded by transducers located closest to the load location—see Figure 10.

**Figure 11:**
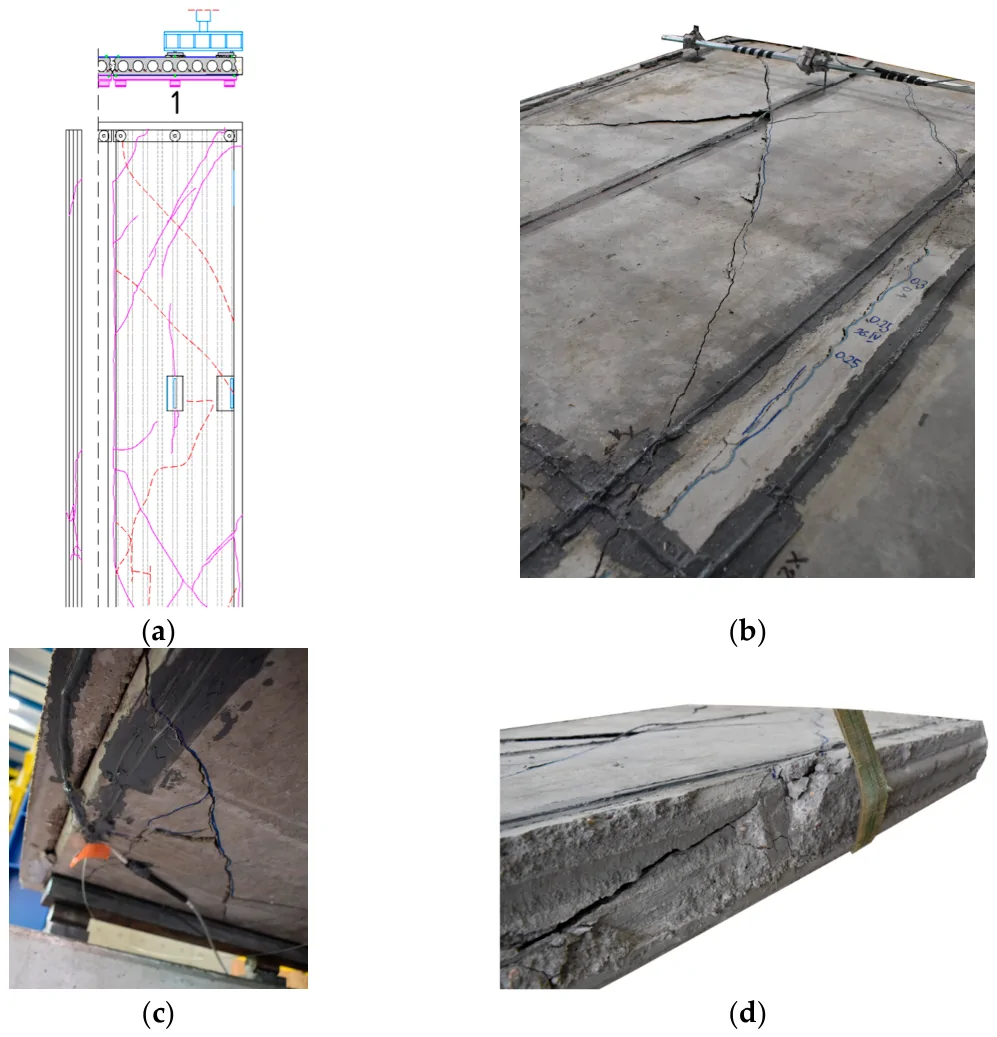
Floor 1 F1: (**a**) sketch of the crack patterns on top (solid magenta lines) and bottom surface (red dashed lines), (**b**) top skew cracking in vicinity of roller supports, (**c**) bottom crack in vicinity of roller support propagating onto outer web, and (**d**) top skew crack propagating onto horizontal crack in the outer web.

In Floor 1, position F1, the first cracks were detected by DFOS on the bottom surface at 85 kN; the cracks were flexural near the element’s midspan. The floor failed at 101.9 kN, the damage was localized in the directly loaded element, and no cracks were observed in the adjacent elements. Figure 11a illustrates the crack patterns observed on the top and bottom surface. Large damage was observed in the vicinity of roller support on both the top and bottom surface—see Figure 11b,c. The overall crack pattern, consisting of skew cracks on both the top and bottom surface, resembled a spiral, torsional cracking, similar to that observed by Pajari on single HC specimens subjected to pure torsion [30]. A large horizontal crack following the joint groove was observed on the surface of the outer web in the center of the floor—see Figure 11d.

**Figure 12:**
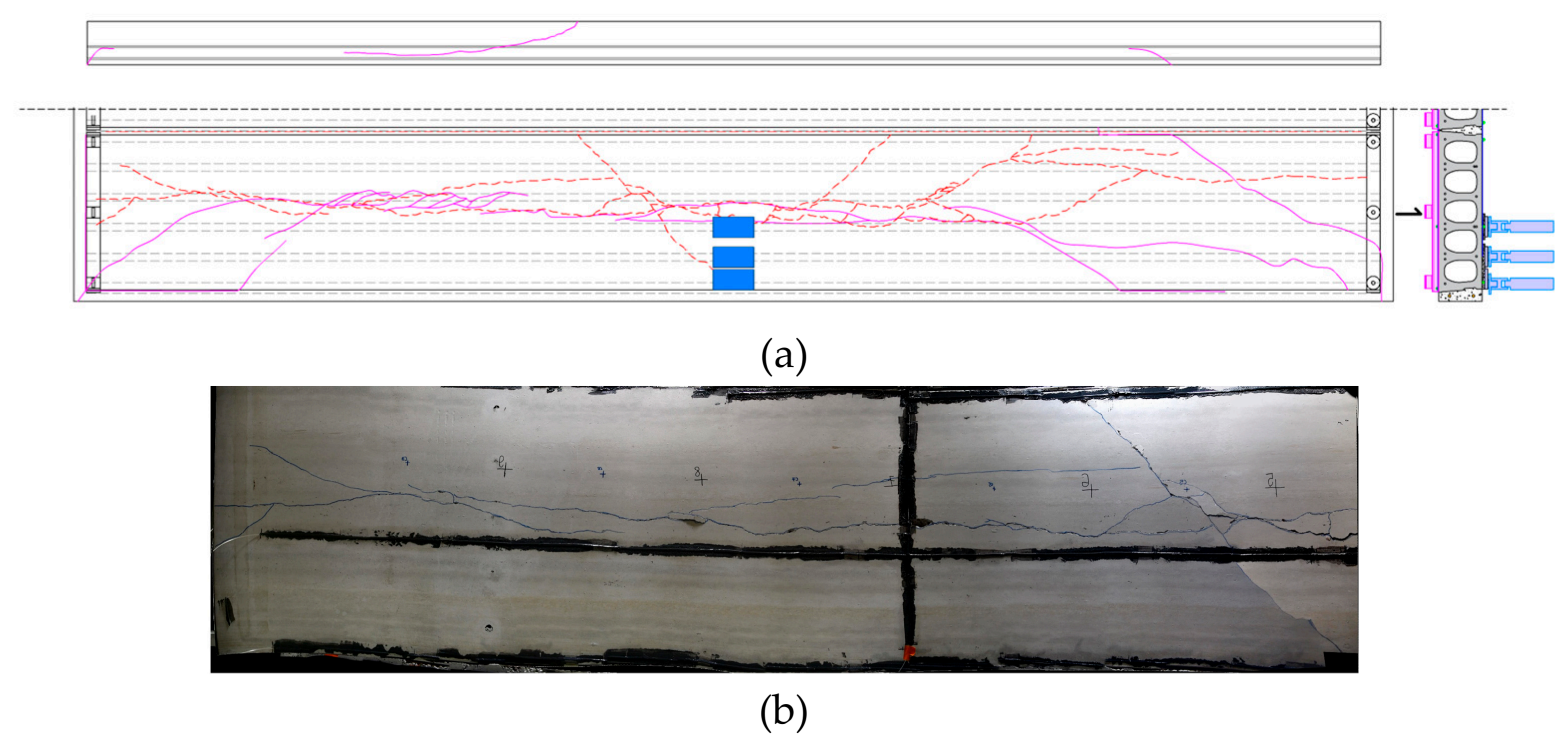
Floor 2 F1: (**a**) sketch of the crack patterns on top (solid magenta lines) and bottom surface (red dashed lines), and (**b**) bottom longitudinal crack with inclined branch near elements’ center.

Floor 2 loaded in position F1 failed in a brittle manner. A sudden crack opening was observed at 228.6 kN without gradual propagation; yet, the onset of bottom flexural cracking was detected by DFOS at 180 kN. The cracks were longitudinal along the entire length of the directly loaded element on both the top and bottom surface, transitioning into skew patterns near supports—see Figure 12a. The damage on the bottom surface was more pronounced; a skew crack was observed near the element’s center—see Figure 12b. The skew bottom crack turned into a shear crack on the outermost web, transitioning into a horizontal crack in the joint groove—see side view on Figure 12a.

**Figure 13:**
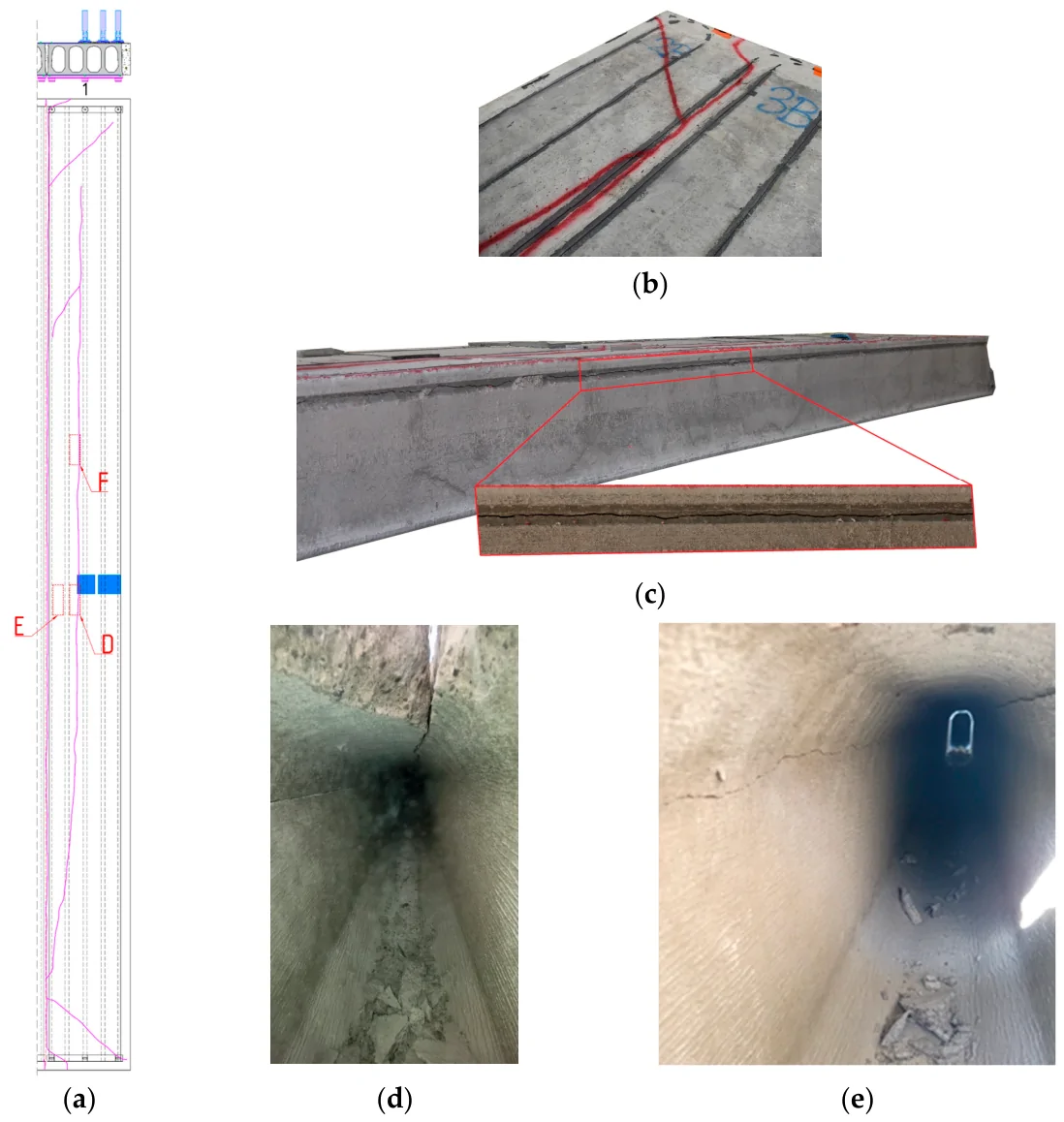
Floor 3 F1: (**a**) sketch of the crack patterns on top (solid magenta lines) and bottom surface (red dashed lines), (**b**) inclination of the top crack in roller support vicinity, (**c**) horizontal crack in the joint groove, (**d**) horizontal crack seen through opening in point “D”—crack in the top flange, and horizontal crack in the top part of the central web, and (**e**) horizontal crack seen through opening in point “E”—horizontal crack in the penultimate (left-hand side) and ultimate (right-hand side) webs.

Floor 3 loaded in position F1 behaved similarly to Floor 2. A sudden longitudinal crack opening was observed on the top, at the force value of 325 kN. No cracking was visually observed on the bottom surface; yet, DFOS detected a single flexural crack opening at approximately 250 kN. The top longitudinal crack extended along the entire length of the directly loaded element and turned into a skew crack towards the floor’s corner near its supports—see Figure 13a,b. The failure was accompanied by a large opening of the joint. After the floor disassembly, a horizontal crack in the joint groove was observed—see Figure 13c. Three openings were made through the top flange, with locations marked as D, E, and F in Figure 13a. The openings uncovered horizontal web cracks—see Figure 13d,e.

**Figure 14:**
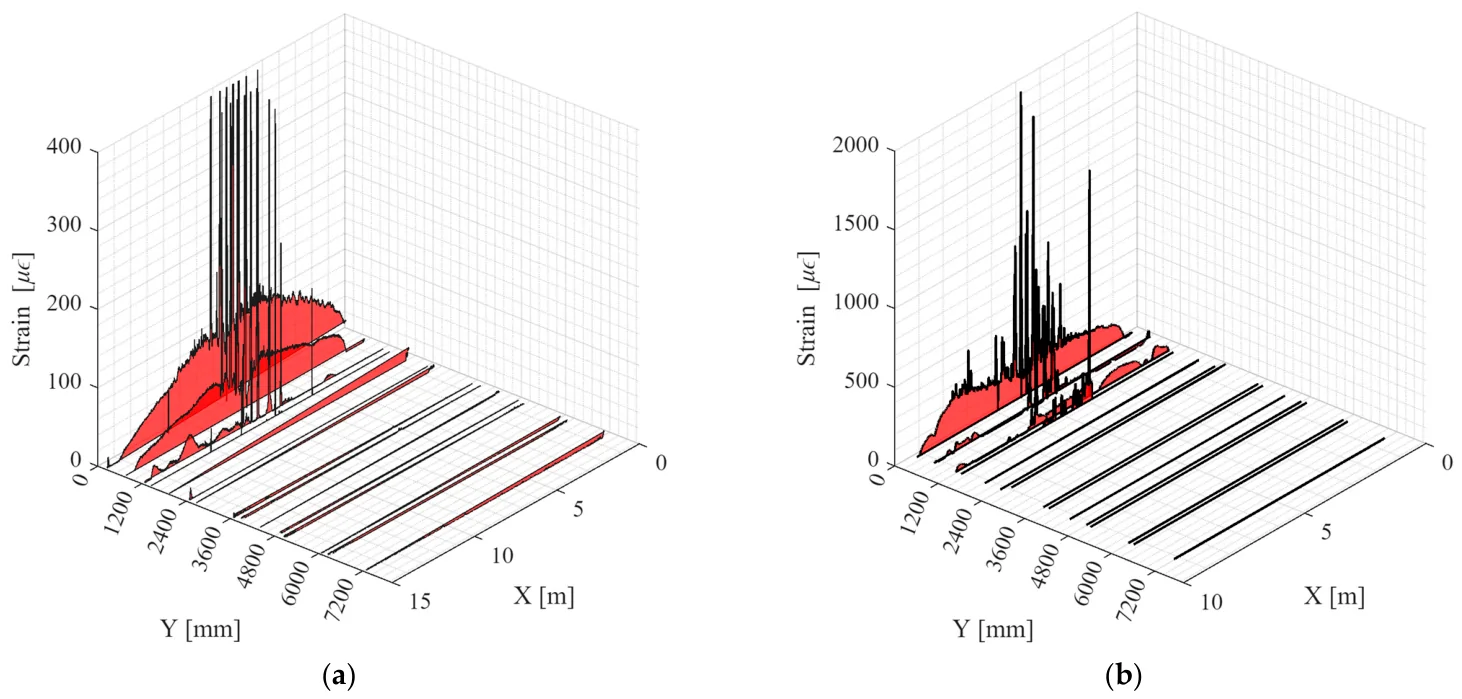
Bottom strain measurements in X direction performed after load in position F1 was removed: (**a**) Floor 2, and (**b**) Floor 3.

In all F1 floor experiments, cracks were visually observed only in the directly loaded outer element. DFOS data collected in Floors 2 and 3 from the dynamic interrogators (providing constant strain measurements, unavailable in Floor 1 on the unloaded elements) after the load was removed confirmed this observation—see selected residual bottom X strain values plotted in Figure 14.

**Figure 15:**
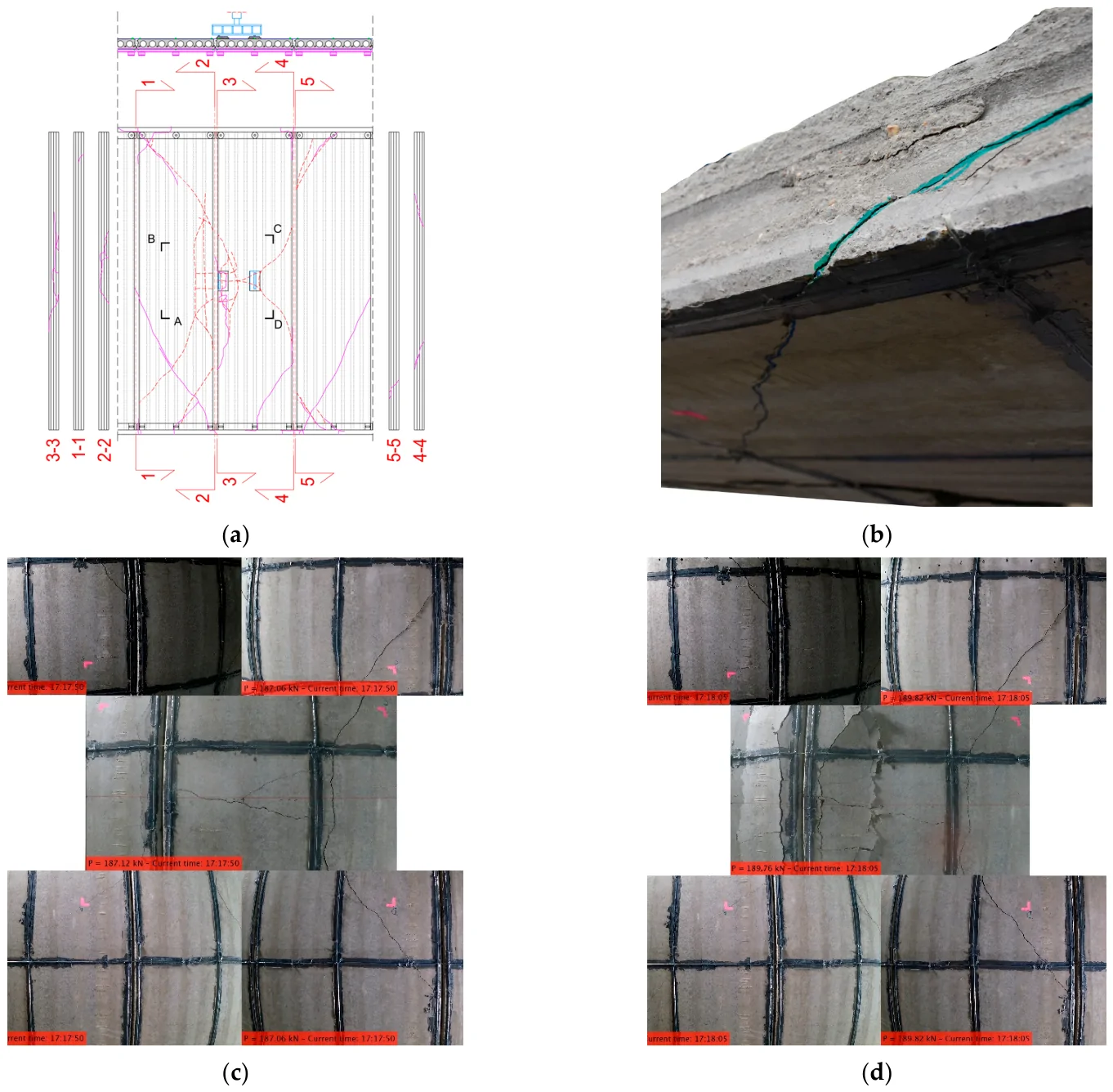
Floor 1 F2: (**a**) sketch of the crack patterns on top (solid magenta lines) and bottom surface (red dashed lines), (**b**) skew bottom crack (blue) propagating onto outer web (green), as sketched on section 4–4, (**c**) flexural and skew cracking observed on the bottom of the specimen at *p* = 187 kN, still from camera, and (**d**) pronounced longitudinal cracking in the directly loaded and adjacent (left-hand side of the central frame) element captured by camera at failure.

**Figure 16:**
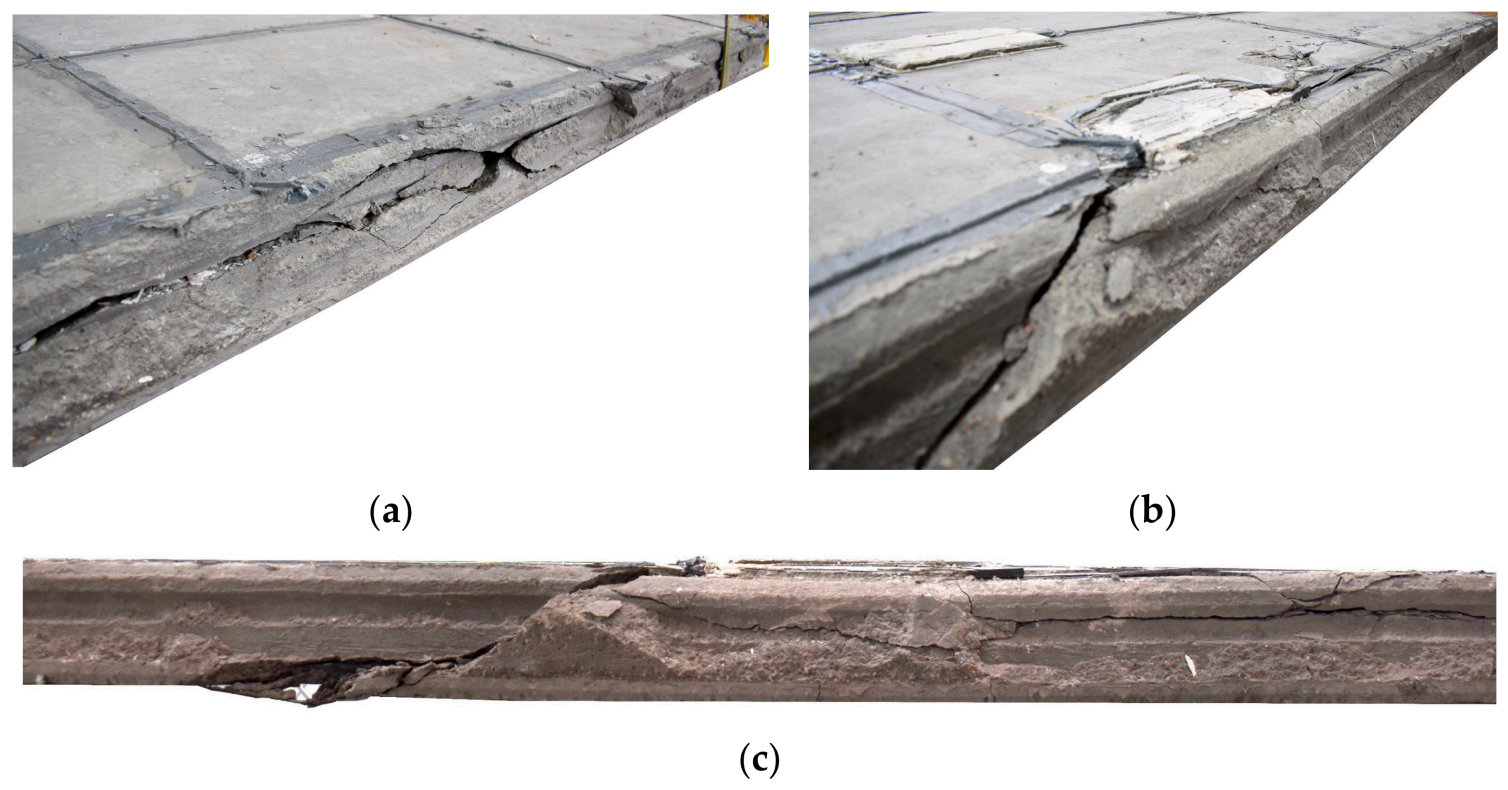
Floor 1 F2, damage observed in outer webs of directly loaded and adjacent slabs: (**a**) horizontal splitting crack observed in outer web of the adjacent slab, as sketched on section 2–2, (**b**) damage around the steel plate of the directly loaded slab, inclined crack in the outer web, and (**c**) horizontal and skew crack in the outer web of the directly loaded slab, as sketched on section 3–3.

In experiment F2, Floor 1, the observed cracks were predominantly skewed on both the top and bottom surface. Figure 15a illustrates the patterns observed after failure. The first flexural cracks were detected by DFOS in the directly loaded element and on its bottom surface at a load of approximately 140 kN. A clear view of the flexural crack turning into a longitudinal and skew pattern was visually observed at 184 kN—see Figure 15c. Top skew cracks near the support of the directly loaded element were first detected by DFOS at approximately 164 kN, shortly before reaching the first unloading at 182 kN (see curve in Figure 10b). A similar skew crack was also observed visually in the adjacent, unloaded element. Failure occurred at 190.4 kN when a sudden, longitudinal crack opening occurred on both the top and bottom of the specimen. A significant damage concentration was observed around the steel plate placed over the outermost web of the directly loaded element and on the bottom of the adjacent element—see Figure 15d and Figure 16a,b. Upon disassembly of the floor, skew cracks were observed on the webs of the directly loaded element, interconnected with cracks on the bottom surface of the specimen (see section 4–4 of Figure 15a,b). Skew web cracks were also observed in the adjacent, unloaded elements (see sections 1–1 and 5–5 of Figure 15a). Large horizontal and skew damage of the outer webs was observed in the directly loaded and adjacent elements—see sections 2–2 and 3–3 of Figure 15a and Figure 16.

**Figure 17:**
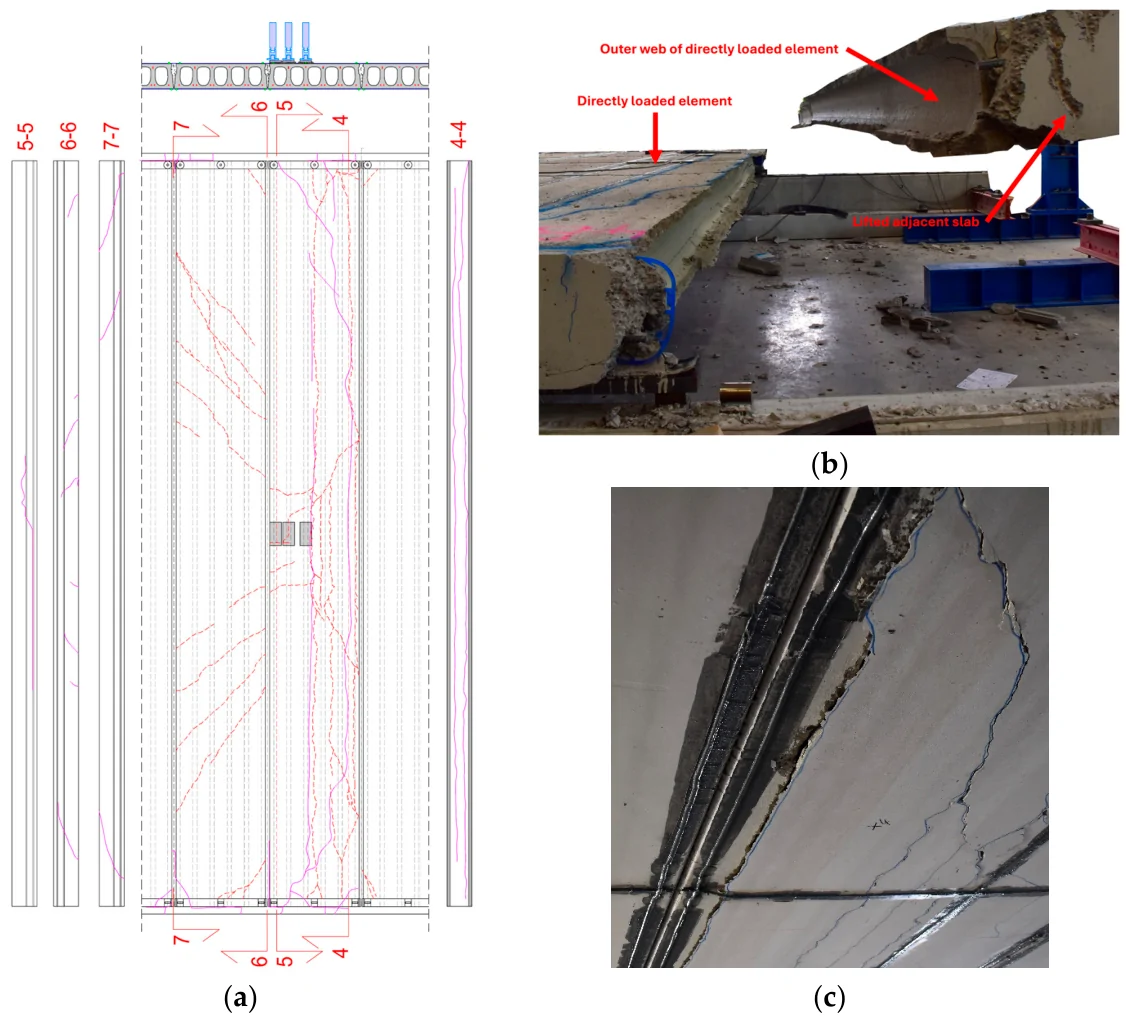
Floor 2 F2: (**a**) sketch of the crack patterns on top (solid magenta lines) and bottom surface (red dashed lines), (**b**) adjacent slab lifted during floor disassembly with visible outer web of the directly loaded element detached from the specimen, and (**c**) close-up picture of the main bottom longitudinal crack in vicinity of the outer web.

**Figure 18:**
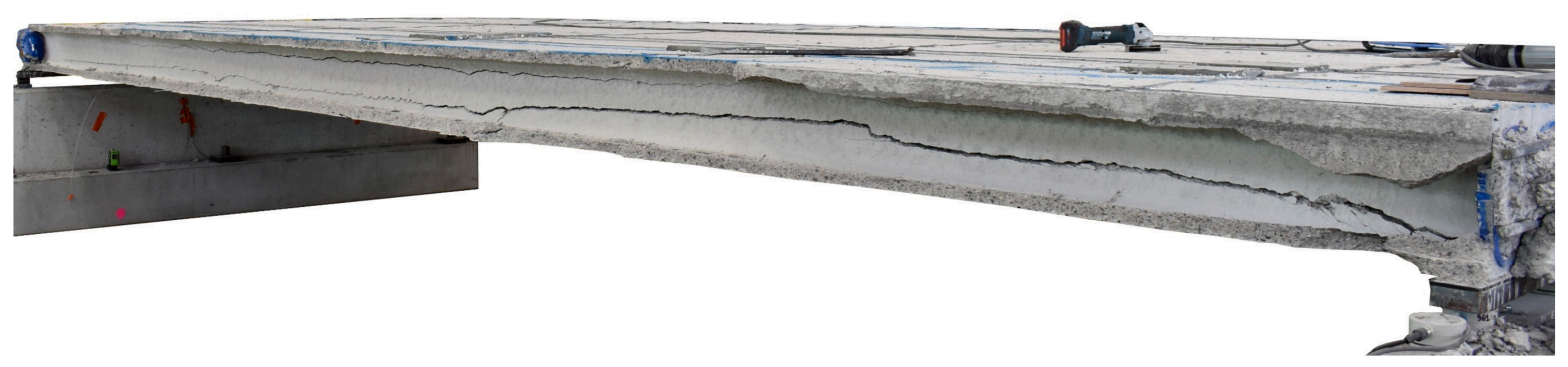
Floor 2 F2: horizontal cracks in the penultimate web of the directly loaded element.

In Floor 2, subjected to the load in position F2, first, flexural cracking was detected by DFOS at 290 kN. A subtle change in stiffness can be seen in the load–displacement curve at this load value—see Figure 10b. The floor failed in a brittle manner, with a sudden opening of longitudinal cracks on both the top and bottom surface at a load equal to 405 kN. A loud noise was heard a few seconds before visual observation was made, signifying possible damage evolution inside the specimen. At a residual value of 295 kN, skew bottom cracks started to develop in the adjacent element; the cracks closed after the load was removed as they were not visible during the post-failure inspection. The patterns were, however, visible in the videos taken from the bottom of the specimen. The top cracks were primarily longitudinal (see Figure 17a), following similar locations as the bottom cracks and transitioning into skew patterns near the supports. The damage on the bottom surface was more pronounced, and the main, widest crack was observed in the vicinity of the outer web of the directly loaded specimen—see Figure 17c. During the disassembly of the floor, the outermost web of the directly loaded element detached from the rest of the specimen due to the significant bottom and top cracking—see Figure 17b. Significant horizontal cracking was uncovered in the penultimate web of the directly loaded specimen—see Figure 18. Horizontal cracks in further webs were also visible through an opening made in the top flange of the directly loaded element. A horizontal crack in the joint groove, similar to the one observed in position F1, was observed in the directly loaded specimen—see section 5–5 in Figure 17a. Inclined cracks were also observed on the surface of the outer webs of the adjacent element—see sections 6–6 and 7–7 in Figure 17a.

**Figure 19:**
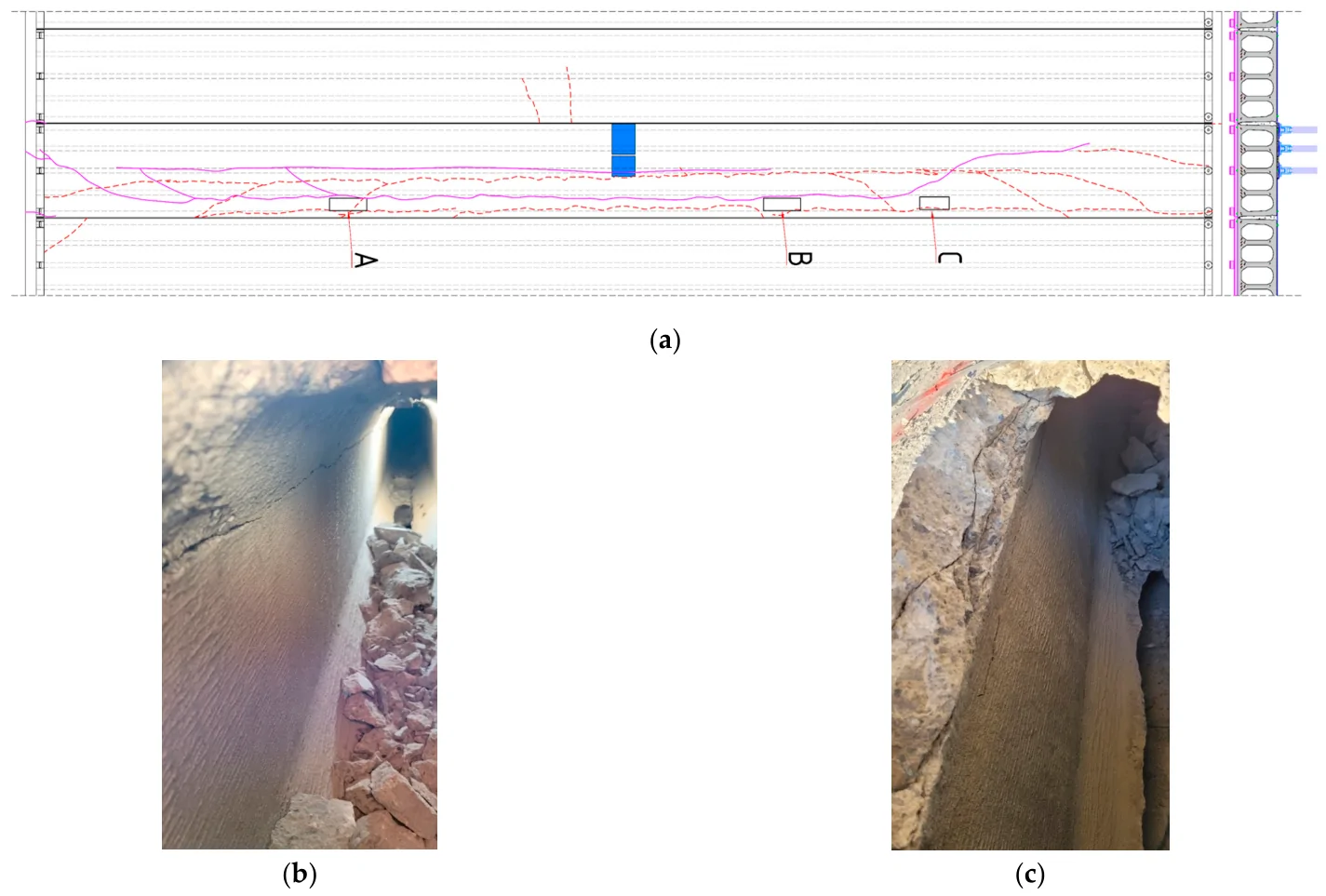
Floor 3 F2: (**a**) sketch of the crack patterns on top (solid magenta lines) and bottom surface (red dashed lines), (**b**) horizontal web crack seen through point “B”, and (**c**) horizontal web crack seen through point “C”.

No gradual crack development was observed visually in Floor 3 subjected to load F2. The linear behavior with a subtle change in stiffness pre-failure can be seen on the load–displacement curve presented in Figure 10b. Failure occurred at 527 kN when a sudden opening of the longitudinal crack was observed in the directly loaded element on both the top and bottom surface—see Figure 19a. The cracks continued throughout the entire length of the specimen, in the vicinity of the central and penultimate web. The widest bottom crack was observed in the vicinity of the ultimate web. After the peak load was reached, a subtle softening occurred, followed by steep unloading to a residual value of approximately 400 kN. Even though no cracks were observed in the adjacent elements, DFOS measured significant strain peaks at the peak force value in the vicinity of the floor midspan in all the elements. The presence of these cracks was not confirmed visually, possibly due to cracks closing upon removing the load. Similarly to load position F1, openings were made in the top flange of the specimen, with locations marked as A, B, and C in Figure 19a. The openings revealed horizontal cracks in the ultimate and penultimate webs of the specimen—see Figure 19b,c.

### 3.3. Material Properties of Concrete

The mechanical properties of concrete used in the hollow-core, joints, and tying beams, obtained from the tests performed on the standard sized specimens (cubes, cylinders, and prisms), are summarized in Table 1 for each experiment separately. The table includes the number of tested specimens *n* and the age of the samples at the time of the experiment (Exp) and at the time of the material property evaluation (Test). The mechanical properties obtained from drilled core specimens are listed in Table 2 in a similar manner to Table 1. Table 3 illustrates the relationship between the mean compressive strength measured on standard and drilled core specimens; Table 4 compares the values of the elasticity modulus. Table 5 compares the re-calculated values of the uniaxial tensile strength of concrete obtained from standard-sized and drilled core specimens. Table 6 illustrates the density of samples.

### 3.4. Numerical Simulations

**Figure 20:**
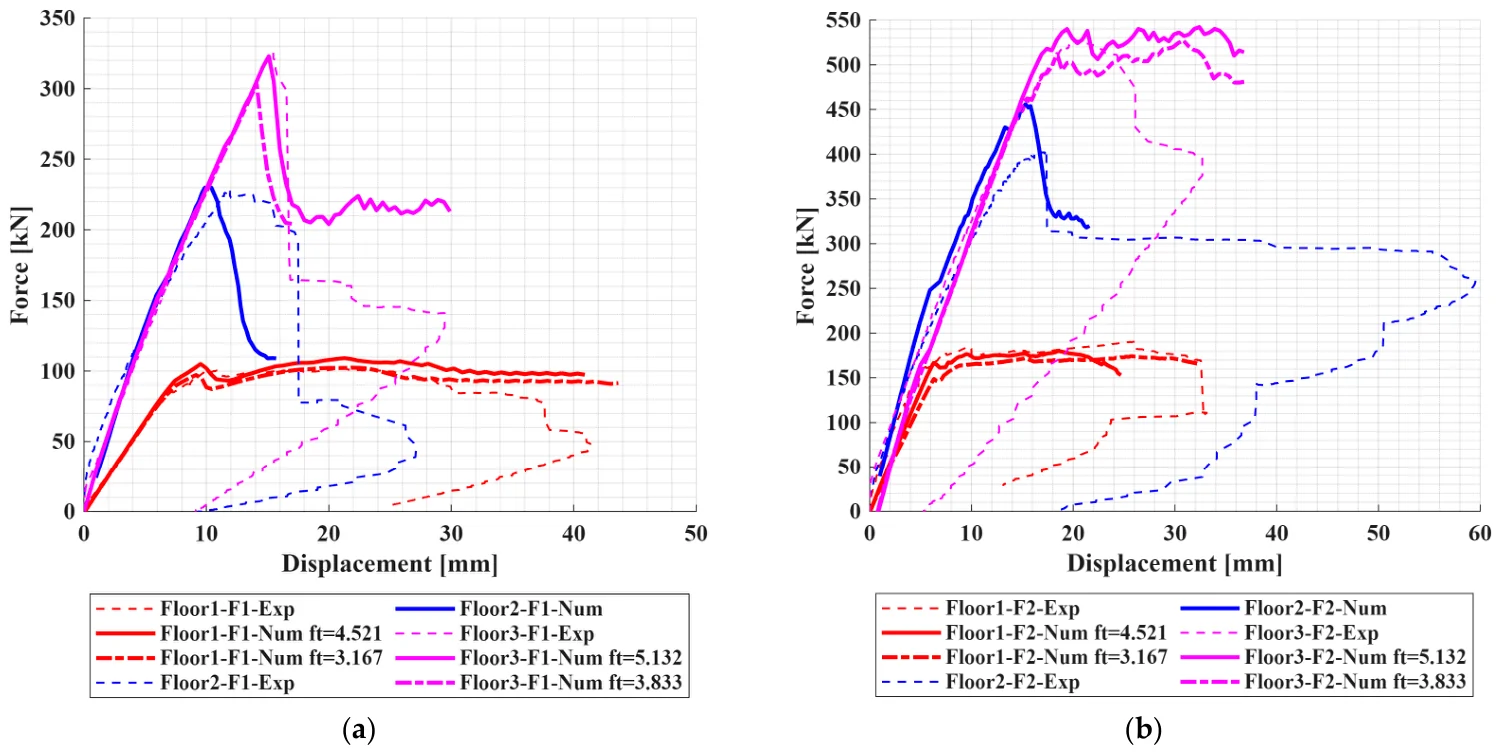
Comparison of load–displacement curves obtained from numerical simulations (solid lines) and experimental data (dashed lines): (**a**) position F1, and (**b**) position F2.

**Figure 21:**
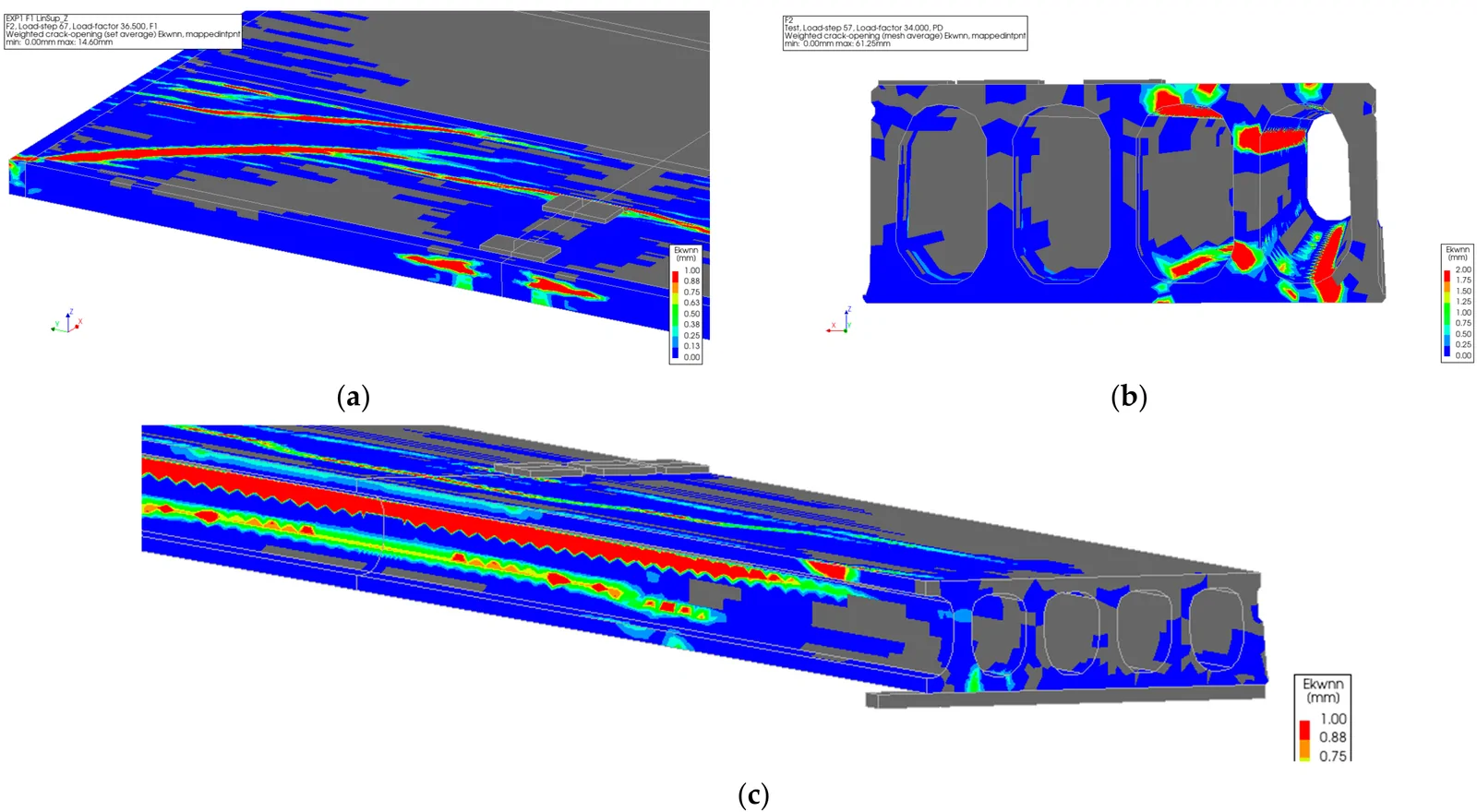
Chosen crack patterns obtained from numerical models: (**a**) perspective view of the skew cracking pattern—Floor 1, F1, (**b**) horizontal cracks in penultimate web, Floor 2, F2, and (**c**) horizontal cracks in penultimate web, Floor 3, F2.

The results of the numerical simulations are illustrated in Figure 20 where the load-displacement curves obtained from the numerical models, prepared with different tensile strengths of concrete, are plotted against the experimental values. A comparison between the peak loads obtained in the experiments and numerical models is presented in Table 7. Figure 21 illustrates selected crack patterns obtained numerically.

## 4. Discussion

The results of the experimental tests, in terms of the peak load at failure P_ult_, displacement at peak load Δ_ult_, cracking load P_cr_, and displacement at cracking load Δ_cr_, as well as peak load enhancement P_ult,Element_/P_ult,Floor_ and cracking load enhancement P_cr,Element_/P_cr,Floor_, are summarized in Table 8 below. In order to provide a quantitative assessment of the ductility of both single specimens and full floors, two simple ductility measures [61] based on measured cracking and ultimate displacements μ_d_ and forces μ_z_ were calculated.

As presented in Figure 6, single specimens behaved in a similar manner. The elements reached significant deflections before failure, with a visible change in post-cracking stiffness along with observed gradual flexural crack development—see Table 8. Finally, an abrupt rupture of the elements caused by flexure (SP150) and shear–flexure (AVU320 and HC500) occurred. Nevertheless, due to the gradual evolution of deformations and cracks that preceded the abrupt failure, the behavior of single specimens, when compared to full floors, can be characterized as ductile, which is also confirmed by the quantitative assessment presented in Table 8.

Since the load was applied at the midspan, the elements were subjected to combined shear, torsion, and flexure; despite that, no cracks associated with torsion were observed, in contrast to the experiments on HC subjected to pure torsion conducted by Pajari [30]. In Pajari’s experiments concerning the shear–torsion interaction [29], where loads were applied in a similar manner but in the vicinity of the support, skew and longitudinal cracks were observed on the top and bottom surfaces of the elements. Such cracks were not observed in SP150 and HC500, whereas longitudinal cracking causing web separation was observed in AVU320—see Figure 8b.

The eccentric load did, however, result in uneven cracking patterns, suggesting that a larger portion of the applied load was carried by the webs closer to the resultant force acting on the specimen. The aforementioned effect was different in all three slabs: in SP150, it had the least impact, as the critical flexural crack propagated across the entire width of the element—see Figure 7; in HC500, an inclined shear crack was observed in both the outer loaded and unloaded webs—yet, both cracks had a different morphology, see Figure 9. The eccentricity effect was most pronounced in AVU320, where the damage was largely localized in the outer loaded web—see Figure 8b.

The full floor specimens subjected to loads applied on the outermost panel (position F1) behaved in a much more brittle manner than the corresponding single elements (compare Figure 6 and Figure 10a); the difference is also clearly visible when the ductility factors are compared—see Table 8. A visible difference in ductility can also be observed between the shallow Floor 1 built of SP150 elements and Floors 2 and 3 built of deep, slender web AVU320 and HC500, as, in the case of the latter, the failure was not preceded by a visible change in stiffness and crack propagation observed in Floor 1. Even though Floor 1 exhibited more ductile behavior, its extent was largely limited as compared to the single element which reached a peak displacement of 85 mm, whereas the floor specimen reached a peak displacement of just 21 mm. The difference is clearly illustrated through ductility factors, which also decrease significantly, albeit to a lesser extent than in the case of Floors 2 and 3.

The change in ductility was more evident in the case of Floors 2 and 3 which failed at peak displacements close to 15 mm compared to 160 reached in the corresponding single-element tests, with ductility factors dropping substantially—see Table 8. In all three experiments, damage was localized in the directly loaded element only. Despite this, in the case of Floors 2 and 3, a significant improvement in capacity was observed, compared to single elements as the peak load obtained in the full floor was 1.41 and 1.49 times higher than the single-element failure load—see Table 8. In the case of Floor 1, the improvement in capacity was negligible, as the floor failed at a load approximately 9% higher. The capacity improvement was therefore not constant even though the floors were of the same scale.

Floor specimens exhibited failure modes distinct from single elements. The failure of Floor 1 was largely governed by torsion, as spatial skew cracks were prevalent (see Figure 11a–c), while a horizontal crack in the joint groove (see Figure 11d) indicates a significant stress concentration in the joint groove, which facilitates the load transfer to adjacent elements. The failure of Floor 2 was governed by transverse flexure, as the main damage pattern consisted of longitudinal cracks in the top and bottom flanges, and the inclination of the cracks in the vicinity of supports and midspan indicates moderate torsional effects—see Figure 12. The mode of failure observed in Floor 3 was similar to that of Floor 2 as the HC500 specimen failed in transverse flexure, with the directly loaded element exhibiting longitudinal cracking in both flanges and webs. Similarly to Floor 1, horizontal joint-groove cracking was observed.

Similarly to position F1, floor specimens subjected to point loads located on the central panel (F2) behaved in a brittle manner compared to corresponding single element experiments—compare Figure 6 and Figure 10b. Similarities to position F1 can also be seen when the response of Floors 2 and 3 are compared to Floor 1, which behaved in a much more ductile manner, exhibiting visible crack propagation and resulting in a ductility factor similar to the single-element test, yet reaching significantly lower displacements—see Table 8. The load–displacement behavior of all three floors was, overall, similar to F1; yet, the specimens behaved in a much stiffer way, reaching peak displacements lower than in the outermost load position.

In contrast to Floor 1, F1, damage was not localized in the directly loaded element, as pronounced skew cracking was observed in two adjacent elements in Floor 1, F2—see Figure 15. Moreover, large damage was observed in the web of the adjacent element as well as on its bottom surface—see Figure 15d and Figure 16. Such damage propagation indicates that the pre-cracked joint was able to effectively transfer loads of significant magnitude. Skew cracks in three elements indicate, similarly to F1, the large contribution of torsion in the failure mode of the floor; however, further damage propagation was limited due to the local failure of the directly loaded and adjacent web. The local, cone-shaped cracks around the steel plate (see Figure 16b) can be attributed to local stress concentrations leading to failure similar to punching observed in solid slabs.

The failure of Floor 2 loaded in position F2 was similar to position F1 as it was mainly governed by transverse flexure. The cracks were primarily longitudinal (see Figure 17a), and the sudden opening of cracks observed on the top and bottom surface of the element was preceded by a loud noise, which could indicate that damage was initiated in the penultimate web of the directly loaded slab, in which extensive horizontal cracking was observed—see Figure 18. In contrast to Floor 2, position F1, cracks were observed in the adjacent element; yet, their opening was not noticed until extensive damage took place in the directly loaded element; the extent and widths of cracks in the adjacent element were significantly smaller.

Floor 3, position F2, failed similarly to the outermost F1 specimen due to transverse flexure. A sudden opening of longitudinal cracks was observed on both the top and bottom surface of the element (see Figure 19a); cracks attributed to transverse flexural behavior were also observed in the specimens’ webs—see Figure 19b,c. Limited cracking was observed visually in the adjacent elements; yet, significant peaks were observed in the DFOS strain measurements performed on the bottom of all the measured specimens, indicating that flexural cracking might have developed throughout the entire width of the floor.

A significant improvement in capacity, when compared to single specimens, was observed in all three experiments in the F2 position. The floors’ capacities were 2.03, 2.51, and 2.41 times higher in Floors 1, 2, and 3, respectively—see Table 8. This indicates a significant improvement in contrast to F1, even though the extent of damage in adjacent elements was limited in the case of Floors 2 and 3. The difference in the floor specimens’ behavior may be attributed to the shape of the HC cross-sections investigated—see Figure 1. The shallow SP150 cross-section with circular voids is characterized by a flexural-to-torsional stiffness ratio of 0.75, meaning that it is more rigid in torsion than in flexure. Moreover, the circular shape of the webs and their insignificant height affect the section transverse stiffness—the SP150 is less sensitive to transverse deformations. In contrast, the webs of AVU320 and HC500 are slender due to the specific shape of the semi-rectangular cores, which causes the sections to undergo large transverse deformations similar to those observed in Vierendeel trusses, with similar effects observed in HC floors supported on flexible supports [62,63,64]. Moreover, AVU320 and HC500 are characterized by flexural-to-torsional stiffness ratios of 0.87 and 1.04, respectively, which can further explain the shift in failure mode.

Previous research on the considered floors in serviceability conditions [48] showed that, in the case of point loads applied on the outermost element, the bending moment in the directly loaded element can be decreased by a significant margin reaching 60, 45, and 35% of the undistributed value in Floors 1, 2, and 3, respectively, while, in the case of the central load position, the decrease reached 40, 30, and 27%. This can explain the difference in behavior when compared to a single element, where flexural effects were of considerable importance, as well as the fact that, despite damage localization in position F1, improvements in capacity were observed. The magnitude of the bending moment distribution is, however, not unambiguously equivalent to an increase in the load-bearing capacity; yet, a similar pattern is observed in both, as floors of greater spans (Floors 2 and 3) were characterized by a larger increase compared to the floor of a significantly shorter span (Floor 1). This observation is in line with the numerical studies performed by the authors [45], in which a pronounced effect of floors’ span on capacity improvement was observed.

The difference between single element and full floor behavior can be further explained by the imposed boundary conditions. The floor specimens were, to a limited extent, restrained on supports, and lateral displacements and rotations were also reduced due to the presence of adjacent slabs and peripheral tying beams. These restraints induce additional confinement that could positively influence the stress state in HC webs and flanges, increasing elements’ concentrated load-bearing capacity.

Local effects caused by stress concentrations were observed, especially in Floor 1, position F2. Local stress concentrations in joint grooves were also observed in all specimens; yet, as the joint primarily ensures load distribution and the groove act as a support of the directly loaded slab in the transverse direction, such stress concentrations are expected regardless of the load arrangement. It should, however, be noted that, in the case of Floor 1 F2, the presence of local effects was preceded by visible crack propagation into further elements and a significant change in floor stiffness, whereas, in the case of failure governed by local effects, it is expected that we would observe a sudden, brittle failure with damage localized only in the close vicinity of the loading point. Considering also the case of other floors, where cracks propagated throughout the length of the element, it can be concluded that, despite their presence, the local stress concentrations were insignificant, as local failure modes observed in previous experiments concerning loads applied on smaller areas [41,42,44] and numerical analyses [45,65] were not observed. This indicates that the chosen load arrangement, in which several webs are loaded simultaneously, largely mitigates the risk of premature, local failure. Despite the brittle behavior of the floors, the specimens remained in place after the experiments, allowing for safe post-failure inspection; moreover, as seen on Figure 10a,b, Floors 2 and 3 preserved a significant residual, post-peak capacity, possibly allowing further loading. The experiments did not result in the uncontrolled, progressive collapse of the entire system, most likely due to the presence of adequate support and peripheral tying.

The values of the material properties of HC concrete were highly dependent on the type of sample investigated. While it is expected that we would obtain a lower compressive strength of concrete when standard cylinders (⌀150 × 300 mm) are used, compared to standard cube samples (150 × 150 × 150 mm), the ratio between the compressive strength of the cylinder and cube samples is expected to be approximately 0.86 [66,67] for concrete of *f_c_*_,*cube*_ = 77–85 MPa, while, in the presented experiments, the observed ratio was significantly lower as it ranged between 0.60–0.72. Compressive strength tests performed on drilled cores were characterized by higher strength values than the ones obtained from standard-sized cylinders, which is expected, as the size is considerably smaller, and, thus, the probability of the presence of weaker areas is smaller. The ratio between standard-sized specimens and scaled, drilled specimens was, however, non-uniform and highly scattered, as the values ranged from 1.09 to 1.42. The ratio between the compressive strength measured on cubes and drilled cores was within 0.65–0.88. Despite the different sizes of the cores, similar values and proportions were obtained in the Floor 1 and 3 specimens, which were also characterized by a similar compressive strength measured on cube specimens.

Core drilling is often used to measure the compressive strength of concrete in existing structures, with much attention paid to this issue in research [68,69,70]. The minimum diameter of cores considered in international standards and research is usually not smaller than 50 mm; however, in the presented study, drilling with such a diameter was not possible due to the specific geometry of the specimens. Disregarding the minimum required size of the drilled specimens, calculations performed according to ACI 214.4R-10 show that the equivalent cylinder compressive strength of concrete was 1.08–1.12 times higher than the measured core values. The obtained results are in stark contradiction with the experimentally obtained results, as the values obtained on standard-sized specimens were generally lower than the drilled core values. In contrast to ACI 214.4R-10, the calculations conducted according to European Standard EN 13791 indicated that the estimated in situ characteristic cube compressive strength of concrete was 0.85–0.96 times lower than the value measured on cores—these results converge well with the direct comparison of the cube results in Floors 1 and 3, where the ratio between the cube strength and drilled core was equal to 0.86 and 0.88, respectively.

The conversion formulae of former versions of British and CEN standards, mentioned in [70], indicate that the ratio of the standard-sized cube strength to the measured core strength is equal to 0.8, while the ratio between the standard-sized cylinder and core is equal to 1.0. The method proposed by the authors of [70] suggests that the standard-sized cylinder strength is 1.17–1.20 times higher than that measured on cores, resulting in overpredicted results, similar to those obtained using ACI214.4R-10.

The values of the elasticity modulus of concrete measured on cores were 0.76–0.89 times lower than those measured on standard specimens—see Table 4. In contrast to the previously mentioned extensive research on the sample size effect on concrete compressive strength, the problem of the elastic modulus has not been widely investigated [71,72]. The latest research [71] showed that the ratio between standard- (150 × 300 mm) and smaller-sized (80 × 160 mm) specimens was equal to 0.88, thus falling within the range of results obtained herein, indicating a similar effect of smaller-sized specimens on the obtained value of the elasticity modulus.

The results of the tensile strength of concrete, converted to a common, uniaxial value, were overall consistent in Floor 2, as an insignificant difference was obtained between the strength measured on prisms and cylinders, while the core value was approximately 7% lower. In Floors 1 and 3, the values were, however, largely divergent—see Table 5. The uniaxial values, recomputed from three-point bending tests, resulted in the highest values; the difference between the re-calculated splitting strength was insignificant in Floor 1, reaching 5% on average. A significant difference was, however, observed between the prism value and the direct, uniaxial test value performed on the 150 × 300 cylinder in Floor 3, as the direct uniaxial strength measured on the standard specimen was 39% lower. The values measured via uniaxial tests on the drilled cores were lower by a similar factor in both specimens, reaching 0.70–0.75 of the prism value.

According to [73], the diameter of the specimens subjected to uniaxial tension tests should lie between 50 and 100 mm for concrete made with an aggregate size up to 8 or 16 mm; the diameter of the investigated samples did not fall into this category. A recent literature study on the direct tensile testing of concrete [74] indicates that, while direct tensile tests produce results closer to the real tensile strength, compared to indirect tests, which tend to overestimate it, the results of direct tests are influenced by numerous parameters, including the specimen dimensions. The same study indicates that, due to the lack of data, the accurate quantification of this effect has not yet been verified.

A visible difference was observed between the density of the drilled-core and standard-sized specimens—see Table 6. The drilled cores were generally less dense than the standard-sized specimens, which could be explained by the production technology of HC, resulting in a less compacted mix compared to standard-sized specimens. Although the difference in percentage terms was not pronounced, as the cores were 8–3% less dense than the cube specimens and approximately 4% less dense than the cylinders, the difference in absolute numbers, i.e., 88 and 102 kg/m^3^ less than the standard sized specimens, might explain the difference in the measured elasticity modulus, as a visible correlation between density and the elasticity modulus was found by Miazgowicz and Domagała [71]. The difference in the density of the cube and core samples could also explain the difference in compressive strength; yet, it does not explain the lower results obtained for standard cylinders.

It should be noted that the core specimens were of significantly different age than the standard-sized specimens. Nevertheless, considering that the samples were of a significant age, well past 28 days, it is assumed that the effect of the sample age was not of primary importance.

Despite differences in the measured mechanical properties of concrete, the presented numerical models which utilized properties obtained from standard-sized specimens were able to accurately predict the floors’ response, leading to an average relative error in the peak load prediction of less than 10%—see Table 7. The models were also able to predict the correct load–displacement behavior, although the model of Floor 3, F2, resulted in an overestimation ductility—see Figure 20. The crack patterns observed in numerical simulations were generally consistent with those observed in experiments. Numerical simulations performed with the tensile strength values obtained from drilled cores resulted in an insignificant difference in the peak load prediction, as the peak load predicted by numerical models decreased by merely 3–6%, despite the fact that the tensile strength measured on drilled cores was 30% and 25% lower in Floors 1 and 3, respectively. This suggests that, in contrast to the linear dependency between the tensile strength and element capacity governed by principal stress (see Equations (1)–(3)), the actual behavior of floors may not be highly sensitive to the tensile strength of concrete. Such behavior can be explained by the fact that the assumption of the cracking load being equal to the failure load is not applicable in floor systems that are able to distribute loads well beyond cracking, in contrast to single, isolated elements whose structural integrity deteriorates suddenly as cracks other than flexural develop and no secondary load path can be achieved.

It should be noted that the numerical models did not include the actual loading sequence, in which the floor was already damaged due to loading in the F1 position, which could affect the floors’ behavior in the F2 experiment. This could explain a slight overestimation of the capacity in the Floor 2 model as well as the higher ductile response in the Floor 3 model. Nevertheless, a good convergence between the capacity prediction of the numerical model and the experimental observations may indicate that pre-existing damage did not significantly impact the results. This observation is in line with the author’s previous numerical analysis [45] of Pajari’s experiments on the 400 mm floor [44], in which the loading sequence was similarly disregarded.

In order to assess the theoretical capacity of the three floors, point load values Pth causing failure, as defined by criteria given in Equations (1), (3)–(6), were calculated, assuming average and characteristic values of the measured mechanical properties of concrete, without considering the load distribution. The results were then compared with the measured experimental failure loads P_ult_, as shown in Table 8 and summarized in Table 9. In Table 10, values of the load distribution factor α, as well as its ultimate limit state value α_ULS_, obtained from European Standard 1168 [19], are compared with the inverse of the peak and cracking load enhancement factors obtained experimentally and listed in Table 8.

As can be seen in Table 9, in the case of all the three floors, the capacity is governed by transverse flexure. The simplified expression (4) provides a safe approximation for all the considered floors and load positions. The prediction is, however, very restrictive, especially in the case of the shallow 150 and 320 sections and central (F2) load position.

The other local capacity criteria, such as punching and joint failure (in the case of position F1) provided overestimated results. The non-local, criteria concerning the shear and shear–torsion interaction generally provided an overestimated capacity, which would result in even larger errors if the distribution factors, listed in Table 9, were used according to [19] to decrease the internal forces in the directly loaded elements. The results presented in Table 9 clearly indicate large discrepancies between the poorly defined [33] load distribution factor in the European Standard [19] and the experimentally measured cracking and ultimate load enhancement ratios.

It should also be noted that the standard [19] provisions for punching, transverse flexure, and joint capacity are rather simplified, as it they do not properly consider the effect of the load size, and its transverse position, as well as load distribution. Previous numerical research conducted by the authors [45] showed that, despite the possibility of local failure under point loads, the floor geometry as well as the load position significantly affect the load-bearing capacity.

## 5. Conclusions

An extensive experimental program conducted on both single hollow-core specimens and full floor assemblies provided valuable, novel insight into the behavior of precast floor systems of full scale, which are widely applied in practice, yet sparsely investigated in research. The complexity of the investigated problem is multidimensional, as the behavior of HC floors is not only affected by the boundary conditions, floor layout, and its geometry, but also by the specific shape and production process, which influence the mechanical response at both the cross-sectional and material levels. The use of full-scale specimens demonstrates that phenomena occurring in floor assemblies are unlikely to occur in single specimens and, therefore, the relevance of the presented research for both scientific and practical applications is highly valuable. The presented results and their subsequent analysis led the authors to formulate the following conclusions:

- Single-element HC tests showed that torsion from load eccentricity and local stress concentrations have little influence on the load-bearing capacity under midspan loading. Failure is mainly governed by flexure and shear when loads are distributed across multiple webs, unlike the support-region tests of Pajari [29,30] and McGinnis [25].
- The effects of torsion, transverse flexure, and shear, as well as local stress concentrations, cannot be neglected in full floors, which exhibit behavior significantly different from that of single specimens. While single specimens behave in a ductile manner, full floors are much stiffer, which leads to brittle behavior.
- For the investigated specimens, the observed failure mode was influenced by the cross-sectional geometry. The investigated deep elements with semi-rectangular cores and slender webs exhibited a greater susceptibility to transverse flexural effects associated with Vierendeel action. The resulting web cracking resembles that observed in HC floors on flexible supports [62,63,64]. In contrast, shallow elements with round cores are less sensitive to transverse flexure, and their failure is primarily governed by torsion.
- Local stress concentrations can be mitigated by distributing the load over a larger area or across multiple webs, preventing the local punching failures reported in earlier studies [31,41,42,44]. This is consistent with the authors’ previous numerical findings [45,60]. Nevertheless, because load transfer is primarily governed by shear transmission through the joint groove, stress concentrations within the groove remain unavoidable.
- Floor assemblies subjected to concentrated loads can achieve substantially higher capacities than single HC elements. The improvement depends strongly on the transverse load position, being more pronounced for centrally loaded floors than for edge-loaded ones. At the outermost load position, the capacity increased even though damage was observed only in the loaded element. This enhancement is attributed to the confinement provided by tying systems and to the redistribution of bending moments in the loaded element evidenced in [48]. As the degree of the moment redistribution varies with load position, it partly explains the observed capacity differences. However, the increase in capacity cannot be explained by moment redistribution alone and may also depend on the cross-sectional geometry and span length.
- Despite the brittle and sudden failure of the floor specimens, the complete, uncontrolled progressive collapse of the floor did not occur and some of the specimens retained a significant residual capacity. This can be attributed to the proper design of the continuous tying system, which helped maintain the overall structural integrity of the floor.
- The mechanical properties of concrete in HC elements can be strongly influenced by production-related factors. In particular, the significant differences in density observed between drilled cores and standard specimens may indicate variations in concrete compaction, although the compaction quality was not directly assessed in this study. Similar discrepancies were observed in the measured mechanical properties. While compressive strength could be related to cube strength using established conversion factors [70], no reliable correlation was found for the modulus of elasticity or tensile strength. The limited knowledge on specimen size effects, combined with the small number of tested samples and differences in the testing age, prevents firm conclusions and highlights the need for further research, as drilled cores provide a direct representation of the size effects in thin-walled HC elements and of production-related factors affecting concrete properties.
- Nonlinear finite element analysis using volumetric elements and shell interfaces, as described in the authors’ previous work [45], accurately predicted the failure mode and capacity of HC floors. The good fit between experimental results presented herein as well as with the previous tests presented in [45] confirm the applicability of the approach to different cross-sectional geometries, supporting its use in further parametric studies. Simulations with varying concrete tensile strengths, the governing material property in complex HC stress states, indicated that floor assemblies are less sensitive to tensile strength than single elements. This suggests that, despite the concerns related to HC production [21], the analysis and design of HC floors based on properties obtained from standard-sized specimens can be considered reliable. Unlike isolated elements, HC floors subjected to concentrated loads maintain their integrity well beyond the onset of non-flexural cracking due to the load redistribution and restraint provided by adjacent interconnected elements. However, given the limited number of specimens tested, further research is required to fully assess the influence of tensile strength on the HC floor capacity.
- For the tested specimens, an analysis of the current code provisions of European Standard 1168 [19] suggests that the simplified expression limiting the point load resistance due to transverse flexure can significantly underestimate the floor load-bearing capacity, leading to a conservative design. The expression is also limited in that it does not account for the load size, floor geometry, or load position, all of which were shown in this and previous research [45] to significantly affect the structural response. Conversely, the load distribution factors specified in the Standard [19], when applied to non-local failure criteria such as shear, can substantially overestimate the floor point-load capacity. Further research is therefore needed to develop more accurate design provisions that provide both safe and economical guidance for a wide range of point-loading scenarios in hollow-core floors.

## Figures and Tables

**Table 1:** Mechanical properties of concrete—standard-sized specimens.

Experiment	Part	Property	n	Age [Days]		Value ± σ	Unit
				Exp	Test		
Floor 1 (SP150)	HC	fc,cube	6	90	91	82.28 ± 2.26	[MPa]
		fc,cyl	3	90	94	50.13 ± 5.38	[MPa]
		Ec	6	90	94	44.98 ± 2.38	[GPa]
		fct,sp	3	90	94	4.77 ± 0.50	[MPa]
		fct,fl	6	90	94	7.52 ± 0.42	[MPa]
	Joint	fc,cube	3	26	27	20.16 ± 1.12	[MPa]
		Ec	2	26	30	24.00 ± 1.70	[GPa]
		fct,sp	2	26	30	2.39 ± 0.00	[MPa]
	Beam	fc,cube	3	24	25	23.62 ± 2.41	[MPa]
		Ec	2	24	28	24.00 ± 1.70	[GPa]
		fct,sp	2	24	28	2.07 ± 0.29	[MPa]
Floor 2 (AVU320)	HC	fc,cube	6	104	105	77.89 ± 1.20	[MPa]
		fc,cyl	3	104	109	46.42 ± 10.25	[MPa]
		Ec	6	104	109	47.58 ± 2.99	[GPa]
		fct,sp	3	104	109	4.80 ± 0.48	[MPa]
		fct,fl	5	104	106	7.15 ± 0.51	[MPa]
	Joint	fc,cube	3	21	22	31.48 ± 4.57	[MPa]
		Ec	2	21	26	31.05 ± 4.05	[GPa]
		fct,sp	2	21	26	2.11 ± 0.17	[MPa]
	Beam	fc,cube	6	19	20	25.69 ± 0.85	[MPa]
		Ec	3	19	24	29.40 ± 0.86	[GPa]
		fct,sp	3	19	24	2.53 ± 0.05	[MPa]
Floor 3 (HC500)	HC	fc,cube	6	157	159	85.71 ± 2.40	[MPa]
		fc,cyl	1	157	159	61.90 ± 0.00	[MPa]
		Ec	2	157	160	40.55 ± 0.45	[GPa]
		fct	3	157	160	3.12 ± 0.14	[MPa]
		fct,fl	6	157	159	8.54 ± 0.46	[MPa]
	Joint	fc,cube	3	21	23	21.27 ± 0.93	[MPa]
		Ec	3	21	24	27.27 ± 2.54	[GPa]
		fct,sp	3	21	24	1.84 ± 0.21	[MPa]
	Beam	fc,cube	3	17	19	14.09 ± 1.54	[MPa]
		Ec	3	17	20	23.20 ± 1.23	[GPa]
		fct,sp	3	17	20	1.36 ± 0.09	[MPa]
Single–SP150	HC	fc,cube	3	55	55	77.00 ± 1.43	[MPa]
Single-AVU320	HC	fc,cube	3	33	33	69.20 ± 1.08	[MPa]
Single-HC500	HC	fc,cube	3	56	56	84.80 ± 2.94	[MPa]

**Table 2:** Mechanical properties of concrete—drilled-core specimens.

Experiment	Part	Property	n	Age [Days]		Value ± σ	Unit
				Exp	Test		
Floor 1 (SP150)	HC	fc,core	3	90	135	71.10 ± 1.93	[MPa]
		Ec,core	3	90	135	34.23 ± 0.33	[GPa]
		ft,core	3	90	136	3.17 ± 0.12	[MPa]
Floor 2 (AVU320)	HC	fc,core	6	104	146	50.53 ± 3.53	[MPa]
		Ec,core	6	104	146	37.85 ± 1.32	[GPa]
		ft,core	6	104	146	4.03 ± 0.32	[MPa]
Floor 3 (HC500)	HC	fc,core	12	157	230	75.73 ± 7.53	[MPa]
		Ec,core	6	157	230	36.20 ± 0.28	[GPa]
		ft,core	6	157	228	3.83 ± 0.26	[MPa]

**Table 3:** Relationship between compressive strength measured on various samples.

Experiment	fc	Sample	Sample		
	[MPa]		Cube	Cylinder	Core
Floor 1 (SP150)	82.28	Cube	1.000	0.609	0.864
	50.13	Cylinder	1.641	1.000	1.418
	71.1	Core	1.157	0.705	1.000
Floor 2 (AVU320)	77.89	Cube	1.000	0.596	0.649
	46.42	Cylinder	1.678	1.000	1.089
	50.53	Core	1.541	0.919	1.000
Floor 3 (HC500)	85.71	Cube	1.000	0.722	0.884
	61.90	Cylinder	1.385	1.000	1.223
	75.73	Core	1.132	0.817	1.000

**Table 4:** Relationship between elasticity modulus measured on various samples.

Experiment	Ec	Sample	Sample	
	[GPa]		Cylinder	Core
Floor 1 (SP150)	44.98	Cylinder	1.000	0.761
	34.23	Core	1.314	1.000
Floor 2 (AVU320)	47.58	Cylinder	1.000	0.796
	37.85	Core	1.257	1.000
Floor 3 (HC500)	40.55	Cylinder	1.000	0.893
	36.20	Core	1.120	1.000

**Table 5:** Relationship between uniaxial tensile strength measured on various samples.

Experiment	ft	Sample	Sample		
	[MPa]		Prism	Cylinder	Core
Floor 1 (SP150)	4.521	Prism ^1^	1.000	0.950	0.701
	4.294	Cylinder ^2^	1.053	1.000	0.738
	3.167	Core ^3^	1.428	1.356	1.000
Floor 2 (AVU320)	4.301	Prism ^1^	1.000	1.004	0.938
	4.320	Cylinder ^2^	0.996	1.000	0.934
	4.033	Core ^3^	1.066	1.071	1.000
Floor 3 (HC500)	5.132	Prism ^1^	1.000	0.608	0.747
	3.120	Cylinder ^4^	1.645	1.000	1.229
	3.833	Core ^3^	1.339	0.814	1.000

^1^—$f_{t}=0.601\bar{f_{\mathrm{ct},\mathrm{fl}}}$; ^2^—$f_{t}=0.90\bar{f_{\mathrm{ct},\mathrm{sp}}}$; ^3^—$f_{t}=\bar{f_{t,\mathrm{core}}}$; ^4^—$f_{t}=\bar{f_{\mathrm{ct}}}$.

**Table 6:** Density of cube, cylinder, and drilled-core samples.

**Experiment**	**Sample**	**Density ± σ**
		**[kg/m^3^]**
Floor 1 (SP150)	Cube	2415 ± 8
	Cylinder	2372 ± 13
Floor 2 (AVU320)	Cube	2351 ± 11
	Cylinder	2368 ± 11
	Core	2280 ± 13
Floor 3 (HC500)	Cube	2441 ± 64
	Cylinder	2355 ± 5
	Core	2253 ± 7

**Table 7:** Summary of peak loads registered in experiments and obtained in numerical simulations.

Experiment	Position	Force [kN]			Prediction Error [%]	
		Exp	Num-a ^1^	Num-b ^2^	Num-a ^1^	Num-b ^2^
Floor 1	F1	101.90	109.20	102.35	7.16	0.44
	F2	190.40	180.00	174.31	5.78	9.23
Floor 2	F1	228.60	230.00	n/a	0.61	n/a
	F2	405.20	456.00	n/a	12.54	n/a
Floor 3	F1	325.80	323.00	302.91	0.87	7.56
	F2	527.00	542.00	527.23	2.85	0.04
				$\bar{X}$ =	4.97	4.32
				σ =	4.14	4.12

^1^—$f_{t}=0.601\bar{f_{\mathrm{ct},\mathrm{fl}}}$; ^2^—$f_{t}=\bar{f_{t,\mathrm{core}}}$.

**Table 8:** Summary of experimental results obtained on single and full floor specimens, and estimation of the single element and full floor specimens’ ductility, peak, and cracking load enhancement ratio.

Section	Experiment	P_ult_	Δ_ult_	P_ult,single_/P_ult,Floor_	P_cr_	Δ_cr_	P_cr,single_/P_cr,Floor_	μ_d_ ^1^	μ_z_ ^2^
		[kN]	[mm]		[kN]	[mm]			
SP150	Single	93.7	84.9	-	65.0	14.0	-	5.1	8.7
	Floor-F1	101.9	21.3	1.09	85.0	7.4	1.31	1.9	3.4
	Floor-F2	190.4	26.0	2.03	140.0	4.2	2.15	5.2	8.4
AVU320	Single	161.6	106.1	-	90.0	18.6	-	4.7	10.2
	Floor-F1	228.6	14.0	1.41	180.0	9.5	2.00	0.5	1.9
	Floor-F2	405.2	17.3	2.51	290.0	9.5	3.22	0.8	2.6
HC500	Single	218.1	161.1	-	110.0	19.2	-	7.4	16.6
	Floor-F1	325.8	15.5	1.49	250.0	11.2	2.27	0.4	1.8
	Floor-F2	527.0	20.4	2.42	311.0	9.4	2.83	1.2	3.7

^1^—μ_d_ = (Δ_ult_ − Δ_cr_)/Δ_cr_; ^2^—μ_z_ = (P_ult_/P_cr_)/(Δ_ult_/Δ_cr_).

**Table 9:** Theoretical point-load bearing capacity of the three HC cross-sections compared with experimental results.

Capacity Criterion	Pth [kN]			Pth/P_ult_					
	Floor 1	Floor 2	Floor 3	Floor 1		Floor 2		Floor 3	
				F1	F2	F1	F2	F1	F2
(5) Punching	290.0	630.8	1084.0	2.85	1.52	2.76	1.56	3.33	2.06
(4) Transverse flexure	*36.1*	*123.9*	*236.1*	*0.35*	*0.19*	*0.54*	*0.31*	*0.72*	*0.45*
(6) Joint failure—F1	200.8	438.2	500.9	1.97	-	1.92	-	1.54	-
(6) Joint failure—F2	89.6	190.1	287.2	-	0.47	-	0.47	-	0.54
(1) Shear–Tension	319.4	623.1	580.4	3.13	1.68	2.73	1.54	1.78	1.10
(3) Shear–Torsion Interaction *	121.4	559.0	515.3	1.19	0.64	2.45	1.38	1.58	0.98

*—calculated in the prestressing transmission zone.

**Table 10:** EN 1168 distribution factors α compared to experimentally obtained cracking and capacity factors.

Section	Experiment	P_cr,Floor_/P_cr,Single_	P_ult,Floor_/P_ult,Single_	α	α_ULS_
SP150	Floor-F1	0.76	0.92	0.47	0.59
	Floor-F2	0.46	0.49	0.30	0.38
AVU320	Floor-F1	0.50	0.71	0.29	0.36
	Floor-F2	0.31	0.40	0.22	0.28
HC500	Floor-F1	0.44	0.67	0.24	0.30
	Floor-F2	0.35	0.41	0.21	0.26

## Data Availability

The dataset is available upon request from the authors.
